# Dietary intake of plant- and animal-derived protein and incident cardiovascular diseases: the pan-European EPIC-CVD case–cohort study

**DOI:** 10.1016/j.ajcnut.2024.03.006

**Published:** 2024-03-11

**Authors:** Ju-Sheng Zheng, Marinka Steur, Fumiaki Imamura, Heinz Freisling, Laura Johnson, Yvonne T van der Schouw, Tammy YN Tong, Elisabete Weiderpass, Rashmita Bajracharya, Marta Crous-Bou, Christina C Dahm, Alicia K Heath, Daniel B Ibsen, Franziska Jannasch, Verena Katzke, Giovanna Masala, Conchi Moreno-Iribas, Carlotta Sacerdote, Matthias B Schulze, Sabina Sieri, Nicholas J Wareham, John Danesh, Adam S Butterworth, Nita G Forouhi

**Affiliations:** 1 MRC Epidemiology Unit, University of Cambridge School of Clinical Medicine, Cambridge, United Kingdom; 2 School of Life Sciences, Westlake University, Hangzhou, China; 3 Department of Epidemiology, Erasmus Medical Center, University Medical Center, Rotterdam, the Netherlands; 4 International Agency for Research on Cancer (IARC-WHO), Lyon, France; 5 Population Health Sciences, Bristol Medical School, University of Bristol, Bristol, United Kingdom; 6 Julius Center for Health Sciences and Primary Care, University Medical Center Utrecht, Utrecht University, Utrecht, the Netherlands; 7 Cancer Epidemiology Unit, Nuffield Department of Population Health, University of Oxford, Oxford, United Kingdom; 8 Division of Cancer Epidemiology, German Cancer Research Center (DKFZ), Heidelberg, Germany; 9 Unit of Nutrition and Cancer, Cancer Epidemiology Research Program, Catalan Institute of Oncology (ICO) – Bellvitge Biomedical Research Institute (IDIBELL). L'Hospitalet de Llobregat, Barcelona, Spain; 10 Department of Epidemiology, Harvard T.H. Chan School of Public Health. Boston, MA, United States; 11 Department of Public Health, Aarhus University, Aarhus, Denmark; 12 Department of Epidemiology and Biostatistics, School of Public Health, Imperial College London, London, United Kingdom; 13 Steno Diabetes Center Aarhus, Aarhus, Denmark; 14 Department of Nutrition, Sports and Exercise, University of Copenhagen, Copenhagen, Denmark; 15 Department of Molecular Epidemiology, German Institute of Human Nutrition Potsdam-Rehbruecke, Nuthetal, Germany; 16 Institute for Cancer Research, Prevention and Clinical Network (ISPRO), Florence, Italy; 17 Instituto de Salud Pública y Laboral de Navarra, Pamplona, Spain; 18 Centro de Investigación Biomédica en Red de Epidemiología y Salud Pública (CIBERESP), Madrid, Spain; 19 Navarra Institute for Health Research (IdiSNA), Pamplona, Spain; 20 Unit of Cancer Epidemiology, Città della Salute e della Scienza University-Hospital and Center for Cancer Prevention (CPO), Turin, Italy; 21 Institute of Nutritional Science, University of Potsdam, Nuthetal, Germany; 22 Epidemiology and Prevention Unit, Fondazione IRCCS Istituto Nazionale dei Tumori di Milano Via Venezian, Milan, Italy; 23 British Heart Foundation Cardiovascular Epidemiology Unit, Department of Public Health and Primary Care, University of Cambridge, Cambridge, United Kingdom; 24 Victor Phillip Dahdaleh Heart and Lung Research Institute, University of Cambridge, Cambridge, United Kingdom; 25 British Heart Foundation Centre of Research Excellence, University of Cambridge, Cambridge, United Kingdom; 26 National Institute for Health and Care Research Blood and Transplant Research Unit in Donor Health and Behaviour, University of Cambridge, Cambridge, United Kingdom; 27 Health Data Research UK Cambridge, Wellcome Genome Campus and University of Cambridge, Cambridge, United Kingdom; 28 Department of Human Genetics, The Wellcome Sanger Institute, Wellcome Genome Campus, Hinxton, United Kingdom

**Keywords:** plant-derived protein, animal-derived protein, cardiovascular disease, stroke, ischemic heart disease

## Abstract

**Background:**

Epidemiological evidence suggests that a potential association between dietary protein intake and cardiovascular disease (CVD) may depend on the protein source, that is, plant- or animal-derived, but past research was limited and inconclusive.

**Objectives:**

To evaluate the association of dietary plant- or animal-derived protein consumption with risk of CVD, and its components ischemic heart disease (IHD) and stroke.

**Methods:**

This analysis in the European Prospective Investigation into Cancer and Nutrition (EPIC)-CVD case–cohort study included 16,244 incident CVD cases (10,784 IHD and 6423 stroke cases) and 15,141 subcohort members from 7 European countries. We investigated the association of estimated dietary protein intake with CVD, IHD, and stroke (total, fatal, and nonfatal) using multivariable-adjusted Prentice-weighted Cox regression. We estimated isocaloric substitutions of replacing fats and carbohydrates with plant- or animal-derived protein and replacing food-specific animal protein with plant protein. Multiplicative interactions between dietary protein and prespecified variables were tested.

**Results:**

Neither plant- nor animal-derived protein intake was associated with incident CVD, IHD, or stroke in adjusted analyses without or with macronutrient-specified substitution analyses. Higher plant-derived protein intake was associated with 22% lower total stroke incidence among never smokers [HR 0.78, 95% confidence intervals (CI): 0.62, 0.99], but not among current smokers (HR 1.08, 95% CI: 0.83, 1.40, *P*-interaction = 0.004). Moreover, higher plant-derived protein (per 3% total energy) when replacing red meat protein (HR 0.52, 95% CI: 0.31, 0.88), processed meat protein (HR 0.39, 95% CI: 0.17, 0.90), and dairy protein (HR 0.54, 95% CI: 0.30, 0.98) was associated with lower incidence of fatal stroke.

**Conclusion:**

Plant- or animal-derived protein intake was not associated with overall CVD. However, the association of plant-derived protein consumption with lower total stroke incidence among nonsmokers, and with lower incidence of fatal stroke highlights the importance of investigating CVD subtypes and potential interactions. These observations warrant further investigation in diverse populations with varying macronutrient intakes and dietary patterns.

## Introduction

Balanced diet and nutrition are critical for the prevention of cardiovascular disease (CVD), a leading cause of death globally [1,2]. In addition to the widespread public health focus on reducing saturated fat intake and replacing it with polyunsaturated fats for cardiovascular health [3,4], evidence from short-term trials suggests the benefits of replacing carbohydrates with protein for cardiovascular disease risk reduction by promoting weight loss, lowering blood pressure and improving blood lipid levels [5], and reducing inflammation [6]. However, a large prospective study across 18 countries did not support an association for replacing carbohydrates with total protein intake on CVD outcomes [7]. There has been an increasing interest in the potential role of the specific source of protein, plant- or animal-derived, for CVD risk [[8], [9], [10], [11], [12], [13], [14], [15], [16], [17]]. Although currently there is no specific recommendation on plant- or animal-derived protein intake, some dietary guidelines have recently highlighted the importance of a dietary pattern high in plant-based foods, such as beans/legumes, whole grains, nuts/seeds, all of which are food sources of plant-derived protein [18,19]. It remains unclear, however, to what extent the potential health effects of protein may depend on its food sources. Moreover, a so-called “protein transition” – a population shift toward consumption of diets containing considerably more plant-derived protein to replace animal-derived protein – is urgently needed to mitigate climate change [20,21]. Collectively, these factors highlight the importance of disentangling the potential health effects of plant- and animal-derived protein intake, including the replacement of animal-derived protein with plant-derived protein from different food sources.

Specifically for cardiovascular health, the epidemiological evidence regarding associations of plant- or animal-derived protein is inconclusive, ranging from inverse associations of plant-derived protein [8,10,15,22,23] and positive associations of animal-derived protein [8,10,14], to null associations of plant- or animal-derived protein with CVD outcomes [9,16,17]. Past research was limited by issues such as the assessment of only CVD mortality [8] or single CVD outcomes, such as ischemic heart disease (IHD) [9, 10, 17], stroke [16], or the inability to account for the inter-relations between different macronutrients within an isocaloric diet whereby a higher intake of 1 macronutrient (such as protein) would inherently substitute for energy from other macronutrients in the diet at constant levels of energy intake [12,24]. Moreover, interactions of dietary (protein intake) exposure with other factors in relation to CVD risk were also not assessed.

The present study aimed to investigate the associations of plant- and animal-derived protein intake with risk of total CVD, IHD and stroke in a large European prospective study. Our objectives included *1)* accounting for isocaloric substitution for specific macronutrients, as well as for plant protein replacing animal protein, *2)* investigation of these associations for fatal and nonfatal CVD, IHD, and stroke, respectively, and *3)* examination of potential interaction of dietary protein intake with several prespecified factors (such as age, gender, and BMI, kg/m^2^) on the CVD outcomes. As a secondary aim, we also evaluated the cross-sectional association of plant- and animal-derived protein intake with blood lipid concentrations.

## Subjects and Methods

### Study population

The European Prospective Investigation into Cancer and Nutrition (EPIC) study is a prospective cohort including ∼520,000 adult males and females, recruited from 23 centers across 10 European countries between 1992 and 2000 [25]. Participants completed baseline questionnaires on diet, health behaviors, and medical history, as well as a collection of blood samples and measurement of blood pressure, height, and weight. EPIC-CVD is a large, prospective, case–cohort study nested within the EPIC study among participants with a stored blood sample available (*n* = 385,747) [26]. A case–cohort design was implemented in EPIC to enable efficient measurement of molecular factors (e.g., biomarkers of metabolism, genetics) in a reference subcohort that serves as the comparator group for the incidence of several different disease outcomes. The case–cohort design has the advantages of temporal sequence and the power of a cohort study (in that it involves the complete number of incident cases) with the measurement efficiency of a case–control study [27]. Among this eligible population, we ascertained 24,557 cases of incident CVD during the follow-up period. Among the whole EPIC study, a random subcohort of 18,249 center-stratified participants was selected irrespective of future disease status [26]. For this study, we excluded participants with a prior history of myocardial infarction or stroke at baseline (*n* = 9727). We further excluded participants from Norway (*n* = 87) and France (*n* = 632) due to the small sample size and lack of incident CVD cases and excluded participants from Greece due to an unresolved data protection regulation issue (*n* = 1886). We also excluded participants with missing covariates (*n* = 1565). The final sample included 15,141 subcohort members, and 16,244 incident CVD cases (10,784 IHD cases; 6423 stroke cases), among whom 866 incident CVD cases overlapped with the subcohort, as a design feature of a case–cohort study (an average follow-up of 9.6 y, Supplemental Figure 1). Ethical review boards of participating institutions and the International Agency for Research on Cancer approved the study protocol, and all participants provided written informed consent.

### Ascertainment of CVD events

Incident CVD events (nonfatal or fatal IHD or stroke) were defined by codes 410–414 and 430–438 of the International Classification of Diseases Ninth Edition (ICD-9) and codes I20–I25 and I60–I69 of the Tenth Edition (ICD-10) [26]. Individual centers used different methods to ascertain incident outcomes, including self-report and linkage with morbidity or hospital registries. Nonfatal events were further validated by additional review of medical records and/or linkage with registries. Fatal events were generally ascertained through mortality registries. The end of follow-up for the incident events varied between centers and ranged between 2003 and 2010. Nonfatal and fatal events occurring within 28 d of each other were considered a single fatal event. Follow-up data for each participant were censored at the time of the first CVD event or the end of the follow-up period, whichever occurred first.

### Assessment of dietary intake

Habitual dietary food intakes during the past year were assessed by center- and country-specific questionnaires, either self-administered or interviewer administered, as described in detail elsewhere [25]. Nutrient intakes were calculated by multiplying the nutrient content of foods estimated from food composition tables with individually reported or assumed standard portion sizes using the EPIC European Nutrient Database [28]. In the present study, macronutrient intake levels were calculated to represent their percentage of total energy intake (%TEI), where total energy intake was defined as food energy from carbohydrates, protein, and fat but not alcohol as the latter was not considered a feasible “substitution nutrient” to replace protein in the diet. However, in a sensitivity analysis, we also computed energy intake to include alcohol (see below). We divided protein intake into “plant-derived,” “animal-derived,” and “mixed-origin/unknown,” where “mixed-origin/unknown” represented mixed protein sources without clear information on their plant or animal sources [29].

### Measurement of self-reported, biochemical, and anthropometric covariates

Baseline questionnaires were administered including questions regarding past medical history and social and health behavioral factors such as the history of diabetes, hypertension or hyperlipidemia, education, smoking, and physical activity [25]. Serum lipids were measured at Stichting Ingenhousz Laboratory (Etten-Leur, the Netherlands) from samples stored at −196°C. These lipids included total cholesterol (TC), high-density lipoprotein-cholesterol (HDL-C), and triglycerides. Non-HDL-C was calculated as total cholesterol minus HDL-C. Low-density lipoprotein-cholesterol (LDL-C) was estimated based on the Friedewald formula [30]. Height and weight were measured using similar protocols among participants in all EPIC centers, except for the Oxford center in the United Kingdom, where these were self-reported among all participants [25].

### Statistical analyses

All analyses were performed using Stata version 15 (Stata Corporation). We used Bonferroni correction to address the issue of multiple comparisons. Baseline characteristics were evaluated as mean (SD) and *n* (%) among the overall subcohort across gender-specific quintiles of plant- and animal-derived protein intake. The contribution of food groups to plant- and animal-derived protein consumption was assessed in the subcohort by calculating the proportion of plant- or animal-derived protein consumption derived from the respective food groups, overall and in each country separately. Pairwise Spearman correlation coefficients between dietary plant- or animal-derived protein with intake of other macronutrients were calculated across each participating country in the subcohort.

To account for the over-representation of CVD cases in the case–cohort design, Prentice-weighted Cox proportional hazards models were used to estimate hazard ratios (HRs) and 95% confidence intervals (95% CI) of CVD [31], with age as the underlying time variable. Baseline hazards were stratified by gender, and the basic models were adjusted for age at entry, study center, and energy intake. Country-specific HRs (95% CI) were estimated separately and combined in random-effects meta-analysis to obtain pooled effect estimates and 95% CI, and heterogeneity was evaluated using the *I*^2^ statistic. Multivariable adjustment was made for gender (gender-specific baseline hazards), age (continuous, year), study center, energy intake (continuous, kcal/d), education (low, medium, high), physical activity (inactive, moderately inactive, moderately active, active), smoking (current, former, never), alcohol (0–≤6, 6–≤12, 12–≤24, >24, g/d), dietary fiber (continuous, g/d), glycemic index (continuous), BMI (continuous), self-reported history of diabetes (no/unknown, yes), hypertension (no/unknown, yes), and dyslipidemia (no/unknown, yes). Dietary plant- and animal-derived protein intakes were modeled as gender-specific quintiles (derived from the subcohort) to estimate HRs (95% CI) across the quintiles of the protein intake, or as continuous variables to estimate HRs (95% CI) of per 3 %TEI higher consumption using the energy density model [32]. This represented the base model: the interpretation of this model, which includes total energy intake, is for the association of each of plant- or animal-origin protein with endpoints when protein is consumed in place of other energy-bearing macronutrients, that is, fats and carbohydrates (but not alcohol, which is included in the model). Using the above multivariable-adjusted model, potential interactions were evaluated (by adding an interaction term in the model) between plant- or animal-derived protein intake and 9 prespecified variables: age (continuous), gender, BMI (continuous), physical activity, smoking status (current, former, never), glycemic index (continuous), history of diabetes, hypertension, and hyperlipidemia. Stratified analyses based on these predefined characteristics were performed.

In separate isocaloric macronutrient-specific substitution models, the HRs (95% CI) for per 3 %TEI higher plant- or animal-derived protein to replace each macronutrient were estimated by including total energy intake and all macronutrients in the model as continuous variables expressed as %TEI [carbohydrates, saturated fatty acids (SFAs), monounsaturated fatty acids (MUFAs), polyunsaturated fatty acids (PUFAs), mixed/unknown origin protein, and plant- and animal-derived protein], except the nutrient to be “replaced” in the diet [33]. Specifically, we first examined the association of protein intake from 6 animal food groups (red meat, poultry, processed meat, dairy products, fish, and egg intake) with CVD outcomes using the same model as the above main analysis without substitution. Then, we used the substitution models to estimate the HRs (95% CI) (based on the *β* from plant-derived protein) for per 3 %TEI higher energy from plant protein to replace energy from animal protein derived from different food groups, including red meat, poultry, processed meat, dairy products, fish, and egg intake.

Several sensitivity analyses were conducted to test the robustness of the primary multivariable-adjusted models of the association between dietary plant- and animal-derived protein (per 3 %TEI) and CVD outcomes (CVD, IHD, stroke). These included: *1)* computing TEI to include energy from alcohol intake in addition to energy from carbohydrates, fat, and protein; *2)* excluding those with extreme energy intake (including alcohol) <500 or >3500 kcal/d for females or <800 or >4000 kcal/d for males to avoid influence of extreme energy intake; *3)* excluding incident CVD cases occurring within the first 2 y of follow-up to minimize the potential for reverse causality; *4)* excluding those with a self-reported history of diabetes or cancer or with a history of high cholesterol and/or current use of lipid-lowering drugs; *5)* including height as an additional covariate; *6)* excluding glycemic index or dietary fiber as a covariate; *7)* imputing those missing covariates (replacing missing value with mean for continuous variables, and with a dummy indicator for categorical variables); *8)* including blood lipids (HDL-C, non–HDL-C, triglycerides) as additional covariates to test their potential role of effect modification.

We also conducted analyses specifying additional outcomes of fatal and nonfatal CVD, IHD, and stroke, both without and with macronutrient-specific isocaloric substitutions. Moreover, we included total protein intake as an exposure.

Finally, we examined the cross-sectional associations between plant- and animal-derived protein (per 3%TEI) and blood lipids (TC, HDL-C, non–HDL-C, triglycerides, and LDL-C) in the subcohort of the EPIC-CVD study using linear regression, adjusted for the same covariates as the primary analysis with CVD outcomes.

## Results

### Baseline characteristics

Total estimated mean dietary protein consumption was 18.4 (SD, 3.5) %TEI in the subcohort. Animal-derived protein consumption in the subcohort was higher (11.5 (3.7) %TEI) than plant-derived protein consumption (5.4 (1.4) %TEI). Participants with the highest compared with the lowest plant-derived protein intake levels consumed less saturated fat, more dietary fiber, were less likely to be highly educated, and were more likely to have hyperlipidemia and diabetes at baseline; those with the highest compared with the lowest animal-derived protein intake levels consumed less carbohydrates, more alcohol, were more likely to be current smokers and had higher BMI, and less likely to be highly educated (Table 1).

**TABLE 1:** Baseline characteristics by plant- and animal-derived protein intake in the subcohort of the EPIC-CVD study^1^

	Plant-derived protein quintiles					Animal-derived protein quintiles				
	Q1	Q2	Q3	Q4	Q5	Q1	Q2	Q3	Q4	Q5
Plant-derived protein, %TEI	3.62 (0.54)	4.70 (0.23)	5.43 (0.21)	6.18 (0.25)	7.64 (1.00)	5.58 (1.81)	5.37 (1.42)	5.47 (1.41)	5.42 (1.31)	5.33 (1.23)
Animal-derived protein, %TEI	11.5 (4.02)	11.7 (3.7)	11.8 (3.6)	11.8 (3.47)	10.7 (3.7)	6.61 (1.44)	9.26 (0.53)	11.1 (0.52)	13.0 (0.66)	16.8 (2.46)
Age, y	53.4 (9.6)	52.6 (9.4)	51.8 (8.9)	51.8 (8.8)	51.1 (8.6)	50.6 (9.9)	52.1 (9.9)	52.7 (9.0)	53.2 (8.5)	52.2 (8.1)
BMI, kg/m^2^	25.8 (4.1)	26.0 (4.2)	26.1 (4.2)	26.4 (4.3)	26.9 (4.4)	25.0 (4.0)	25.7 (4.1)	26.2 (4.1)	26.5 (4.1)	27.6 (4.4)
Carbohydrate intake, %TEI	45.0 (6.8)	46.4 (6.4)	47.1 (6.3)	47.8 (6.2)	50.1 (6.7)	52.7 (6.2)	49.4 (5.3)	47.5 (5.2)	45.4 (5.2)	41.8 (6.0)
SFA intake, %TEI	17.1 (3.4)	15.3 (2.8)	14.1 (2.8)	12.8 (2.7)	10.7 (2.7)	14.1 (3.9)	14.6 (3.6)	14.3 (3.5)	14.2 (3.4)	13.6 (3.6)
MUFA intake, %TEI	14.8 (3.3)	14.3 (3.5)	14.1 (3.8)	14.2 (4.0)	13.9 (4.1)	12.9 (3.1)	13.8 (3.2)	14.3 (3.6)	14.8 (3.8)	15.5 (4.3)
PUFA intake, %TEI	6.0 (1.9)	6.0 (1.9)	6.0 (2.0)	6.0 (2.1)	6.1 (2.5)	6.0 (2.2)	5.9 (1.9)	5.9 (1.9)	6.0 (2.0)	6.1 (2.3)
Energy intake, kcal/d	2128 (685)	2019 (601)	1946 (597)	1923 (581)	1927 (622)	2044 (682)	2062 (632)	2049 (618)	1977 (577)	1850 (598)
Glycemic index	54.9 (4.1)	55.3 (3.5)	55.8 (3.5)	56.5 (3.6)	58.0 (4.0)	56.5 (3.8)	56.6 (3.6)	56.5 (3.5)	56.0 (3.7)	54.6 (4.3)
Dietary fiber, g/d	18.9 (6.7)	21.6 (7.0)	23.0 (7.3)	24.6 (7.7)	27.4 (8.8)	24.2 (8.4)	23.4 (8.0)	23.5 (8.0)	22.6 (7.7)	21.0 (7.5)
Alcohol, g/d	10.5 (15)	12.5 (17.5)	14.2 (19.4)	14.9 (20.5)	14.8 (20.9)	9.5 (14.1)	11.1 (15.7)	13.4 (18.0)	14.9 (19.8)	17.0 (23.1)
Gender,^2^ % of females	61.0	60.4	61.0	60.8	60.7	61.1	60.8	61.0	60.8	60.2
Physical activity,^2^ %										
Inactive	21.1	20.9	21.9	23.5	28.9	21.7	21.3	21.6	24.6	26.8
Moderately inactive	36.8	34.2	33.1	33.9	31.8	33.5	35.1	34.2	32.8	34.6
Moderately active	23.0	23.2	22.0	21.9	20.5	23.8	22.9	23.1	21.9	19.6
Active	18.1	21.7	23.0	20.7	18.9	21.0	20.7	21.1	20.7	19.1
Smoking,^2^ %										
Never	44.1	47.1	46.4	44.1	49.3	50.8	46.1	45.0	45.9	43.4
Former	27.1	26.8	28.0	28.8	25.4	27.7	29.4	27.6	26.4	25.3
Current	28.8	26.1	25.6	27.2	25.3	21.5	24.5	27.5	27.7	31.3
Education,^2^ %										
Low	39.8	38.1	38.0	45.1	54.5	31.1	39.6	42.6	47.6	51.5
Middle	12.9	13.6	16.3	13.9	12.9	15.4	15.4	14.1	12.5	12.5
High	47.4	48.3	45.7	41.0	32.6	53.5	45.1	43.4	40.0	36.0
Prevalent diabetes,^2^ %	1.7	2.4	2.8	3.5	5.2	2.0	2.4	2.9	3.0	4.8
Prevalent hypertension,^2^ %	19.8	20.5	19.5	20.3	20.4	20.1	21.0	18.8	19.9	20.6
Prevalent hyperlipidemia,^2^ %	10.3	12.5	14.2	16.7	20.5	15.6	12.7	14.5	13.8	16.4

Abbreviations: EPIC-CVD, European Prospective Investigation into Cancer and Nutrition-cardiovascular disease; MUFA, monounsaturated fatty acids; PUFA, polyunsaturated fatty acids; Q, quintile; SFA, saturated fatty acids; TEI, total energy intake.

1 Total sample size for the subcohort is 15,141. %TEI indicates the percentage of energy intake. The table presents the gender-specific quintiles for the plant- or animal-derived protein intake (%TEI).

2 These variables are expressed as %, others are expressed as mean (SD).

The intake distributions varied by country, with highest estimated animal-derived protein intake levels (%TEI) in Spain and lowest levels in Germany; for plant-derived protein intake the highest levels were in Spain and lowest in Sweden (Supplemental Table 1, Supplemental Figure 2). Sources of dietary protein varied markedly by country. Across countries, the largest food source of plant protein was cereal and cereal products, ranging between 45.3% (United Kingdom) and 68.5% (Italy) (Supplemental Table 1). The largest food source of animal protein was dairy and dairy products (33.3 %), followed by red meat (22.7%), fish/shellfish (12.6%), processed meat (12.1%), and poultry (11.8%), varying by country (Supplemental Table 1).

Total protein intake was strongly correlated with animal-derived protein (*r* = 0.91) but not plant-derived protein (*r* = 0.02) (Supplemental Table 2). Plant-derived protein was positively correlated with carbohydrates and negatively correlated with animal-derived protein, SFA, and MUFA. Animal-derived protein was negatively correlated with carbohydrates and positively correlated with SFA and MUFA.

### Associations of dietary protein with CVD, IHD, and stroke

In the multivariable-adjusted base model (with energy adjustment but without macronutrient-specific substitutions), there was no association of plant- or animal-derived protein intake with incidence of CVD, IHD, or stroke in the quartile analyses or dose–response analyses, with low-to-moderate heterogeneity across countries. Per 3%TEI, the HR of CVD was 0.95 (95% CI: 0.84, 1.09) for plant-derived protein and 1.02 (95% CI: 0.99, 1.05) for animal-derived protein (Table 2, Supplemental Figure 3). Sensitivity analyses did not substantially change the results, except that plant-derived protein was inversely associated with CVD, IHD and stroke risk after excluding dietary fiber from the covariate list (Supplemental Table 3).

**TABLE 2:** Prospective associations between dietary intake of plant- and animal-derived protein and incidence of cardiovascular disease^1^

	Quintiles of protein intake					
	Q1	Q2	Q3	Q4	Q5	Per 3% energy
Plant-derived protein						
Median intake, %TEI	3.60	4.71	5.43	6.19	7.68	
CVD (IHD + stroke)						
*N* cases/ total participants	4312/7336	3637/6650	3182/6116	2938/5441	2375/4976	
HR, adjusted for age, gender, center, energy	1.0 (ref)	0.89 (0.77, 1.03)	0.86 (0.76, 0.98)	0.90 (0.71, 1.13)	0.91 (0.74, 1.12)	0.89 (0.76, 1.04)
HR, multivariable-adjusted^2^	1.0 (ref)	0.96 (0.84, 1.09)	0.96 (0.86, 1.07)	0.99 (0.80, 1.21)	0.95 (0.79, 1.15)	0.95 (0.84, 1.09)
IHD						
*N* cases/ total participants	2734 /5851	2369/ 5441	2109/ 5093	1983/ 4642	1679/ 4319	
HR, adjusted for age, gender, center, energy	1.0 (ref)	0.90 (0.76, 1.06)	0.87 (0.74, 1.03)	0.94 (0.72, 1.23)	0.90 (0.70, 1.15)	0.89 (0.75, 1.05)
HR, multivariable-adjusted^2^	1.0 (ref)	0.98 (0.83, 1.15)	0.98 (0.85, 1.13)	1.06 (0.84, 1.34)	0.95 (0.74, 1.22)	0.95 (0.83, 1.09)
Stroke						
*N* cases/total participants	1845/ 5006	1473/ 4605	1253/ 4260	1000/ 3794	852/ 3548	
HR, adjusted for age, gender, center, energy	1.0 (ref)	0.86 (0.78, 0.95)	0.85 (0.76, 0.95)	0.81 (0.69, 0.97)	0.91 (0.77, 1.08)	0.89 (0.76, 1.05)
HR, multivariable-adjusted^2^	1.0 (ref)	0.93 (0.83, 1.05)	0.95 (0.82, 1.08)	0.91 (0.78, 1.08)	0.95 (0.78, 1.17)	0.94 (0.81, 1.08)
Animal-derived protein						
Median intake, %TEI	6.66	9.28	11.1	13.0	16.7	
CVD (IHD + stroke)						
*N* cases/ total participants	2801/ 5599	3024/5742	3311/ 6114	3485/ 6444	3623/ 6620	
HR, adjusted for age, gender, center, energy	1.0 (ref)	0.96 (0.87, 1.06)	1.01 (0.91, 1.11)	0.98 (0.89, 1.08)	1.21 (1.03, 1.43)	1.07 (1.04, 1.10)
HR, multivariable-adjusted^2^	1.0 (ref)	0.92 (0.83, 1.01)	0.93 (0.84, 1.03)	0.89 (0.80, 1.00)	1.06 (0.90, 1.24)	1.02 (0.99, 1.05)
IHD						
*N* cases/ total participants	1881/ 4717	2009/4773	2197/ 5049	2283/ 5318	2414/ 5489	
HR, adjusted for age, gender, center, energy	1.0 (ref)	0.98 (0.89, 1.08)	1.03 (0.93, 1.15)	0.99 (0.89, 1.11)	1.26 (1.11, 1.42)	1.07 (1.04, 1.10)
HR, multivariable-adjusted^2^	1.0 (ref)	0.94 (0.84, 1.05)	0.95 (0.84, 1.06)	0.91 (0.80, 1.03)	1.08 (0.94, 1.25)	1.01 (0.98, 1.05)
Stroke						
*N* cases/ total participants	1111/ 3971	1191/ 4025	1316/ 4216	1391/ 4460	1414/ 4541	
HR, adjusted for age, gender, center, energy	1.0 (ref)	0.90 (0.80, 1.02)	0.97 (0.86, 1.10)	0.95 (0.81, 1.10)	1.12 (0.87, 1.43)	1.05 (1.01, 1.10)
HR, multivariable-adjusted^2^	1.0 (ref)	0.87 (0.77, 0.98)	0.92 (0.81, 1.04)	0.86 (0.75, 0.98)	1.00 (0.79, 1.25)	1.01 (0.98, 1.06)

Abbreviations: CVD, cardiovascular disease; HR, hazard ratio; IHD, ischemic heart disease; Q, quintile.

1 Proportional hazards models were used to estimate hazard ratios (HRs) and 95% confidence intervals (CIs) for quintile 2–5 (Q2–Q5) compared with Q1 and per 3% total energy intake (TEI) higher of plant- or animal-derived protein within each country separately, with age as the underlying time variable. Country-specific HRs (95% CIs) were combined in random-effects meta-analysis to obtain pooled effect estimates and 95% CIs.

2 The multivariable-adjusted hazard ratio (HR) included adjustment for age (years), gender (males, females), center, energy intake (kcal/d), education (low, medium, high), physical activity (inactive, moderately inactive, moderately active, active), smoking (current, former, never), alcohol (0, 0–≤6, 6–≤12, 12–≤24, >24 g/d), dietary fiber (continuous), glycemic index (continuous), BMI (continuous), reported history of diabetes, hypertension, and hyperlipidemia. No specific substitution of energy from other macronutrients was performed in the models.

In the multivariable-adjusted macronutrient-specific isocaloric substitution models, there were no statistically significant associations with CVD, IHD, or stroke incidence for plant- or animal-derived protein when replacing energy from other macronutrients, including SFA, MUFA, PUFA, or carbohydrates (Supplemental Table 4, Supplemental Figures 4–6). In addition, although animal proteins, such as those derived from red meat, were associated with higher risk of incident CVD, IHD, and stroke (Supplemental Table 5), higher plant protein modeled to replace animal protein derived from different food sources was not associated with any of the incident CVD, IHD, or stroke endpoints (Table 3). Moreover, no statistically significant association was observed for total protein intake in models with and without isocaloric substitutions (Supplemental Table 6, Supplemental Figure 7).

**TABLE 3:** Prospective associations between protein consumption and total and subtypes of cardiovascular disease in isocaloric substitution analyses replacing each 3% higher energy intake from animal protein of different food sources with plant-derived protein^1^

	Hazard ratios (95% CIs), multivariable-adjusted^2^					
	Red meat protein	Poultry protein	Processed meat protein	Dairy protein	Fish protein	Egg protein
CVD outcome						
Total CVD	0.87 (0.70, 1.09)	0.99 (0.79, 1.23)	0.88 (0.73, 1.07)	0.95 (0.79, 1.16)	1.04 (0.82, 1.32)	1.15 (0.86, 1.52)
Fatal CVD	0.88 (0.65, 1.19)	1.21 (0.77, 1.91)	0.81 (0.46, 1.42)	1.05 (0.72, 1.53)	1.12 (0.62, 2.01)	0.81 (0.39, 1.68)
Nonfatal CVD	0.88 (0.70, 1.10)	0.96 (0.80, 1.17)	0.88 (0.73, 1.08)	0.94 (0.78, 1.13)	1.03 (0.84, 1.27)	1.20 (0.90, 1.61)
IHD outcome						
Total IHD	0.85 (0.69, 1.06)	0.97 (0.76, 1.25)	0.85 (0.68, 1.05)	0.93 (0.76, 1.13)	1.04 (0.82, 1.32)	1.27 (0.92, 1.73)
Fatal IHD	1.07 (0.76, 1.50)	1.50 (0.82, 2.74)	1.09 (0.57, 2.06)	1.31 (0.92, 1.89)	1.37 (0.73, 2.54)	1.02 (0.49, 2.15)
Nonfatal IHD	0.83 (0.65, 1.04)	0.92 (0.74, 1.14)	0.84 (0.67, 1.04)	0.88 (0.72, 1.07)	1.00 (0.81, 1.23)	1.34 (0.97, 1.85)
Stroke outcome						
Total stroke	0.84 (0.69, 1.03)	0.99 (0.80, 1.23)	0.86 (0.67, 1.11)	0.95 (0.75, 1.19)	1.02 (0.76, 1.35)	1.06 (0.69, 1.63)
Fatal stroke	0.52 (0.31, 0.88)	0.89 (0.50, 1.60)	0.39 (0.17, 0.90)	0.54 (0.30, 0.98)	0.82 (0.34, 1.96)	0.36 (0.10, 1.38)
Nonfatal stroke	0.87 (0.71, 1.07)	1.00 (0.80, 1.24)	0.90 (0.70, 1.17)	0.98 (0.79, 1.21)	1.01 (0.79, 1.28)	1.09 (0.75, 1.60)

Abbreviations: CVD, cardiovascular disease; IHD, ischemic heart disease.

1 Proportional hazards models were used to estimate hazard ratios (HRs) and 95% confidence intervals (95% CIs) for each 3% higher energy intake from plant-derived protein to substitute animal protein from a specific food source within each country separately, with age as the underlying time variable. Country-specific HRs (95% CIs) were combined in random-effects meta-analysis to obtain pooled effect estimates and 95% CIs. The total number of fatal CVD, IHD, and stroke cases was 2587, 1908, and 739, respectively; and number of nonfatal CVD, IHD, and stroke was 14,224, 9158, and 5816, respectively.

2 The multivariable-adjusted HRs (based on the *β* from plant-derived protein) included adjustment for age (y), gender (males, females), center, energy intake (kcal/d), education (low, medium, high), physical activity (inactive, moderately inactive, moderately active, active), smoking (current, former, never), alcohol (0, 0–≤6, 6–≤12, 12–≤24, >24 g/d), dietary fiber (continuous), glycemic index (continuous), BMI (continuous), reported history of diabetes, hypertension, and hyperlipidemia. Substitution model was used in the analyses, where the association of plant-derived protein was estimated by including all macronutrients in the model as continuous variables expressed in % of total energy intake (carbohydrates, saturated fatty acids, monounsaturated fatty acids, polyunsaturated fatty acids, protein from red meat, poultry, processed meat, dairy, fish, and egg, protein from other animal sources, and plant-derived protein), except the animal protein from certain food source to be “replaced” by plant-derived protein.

### Interactions between protein intake and prespecified variables

**FIGURE 1:**
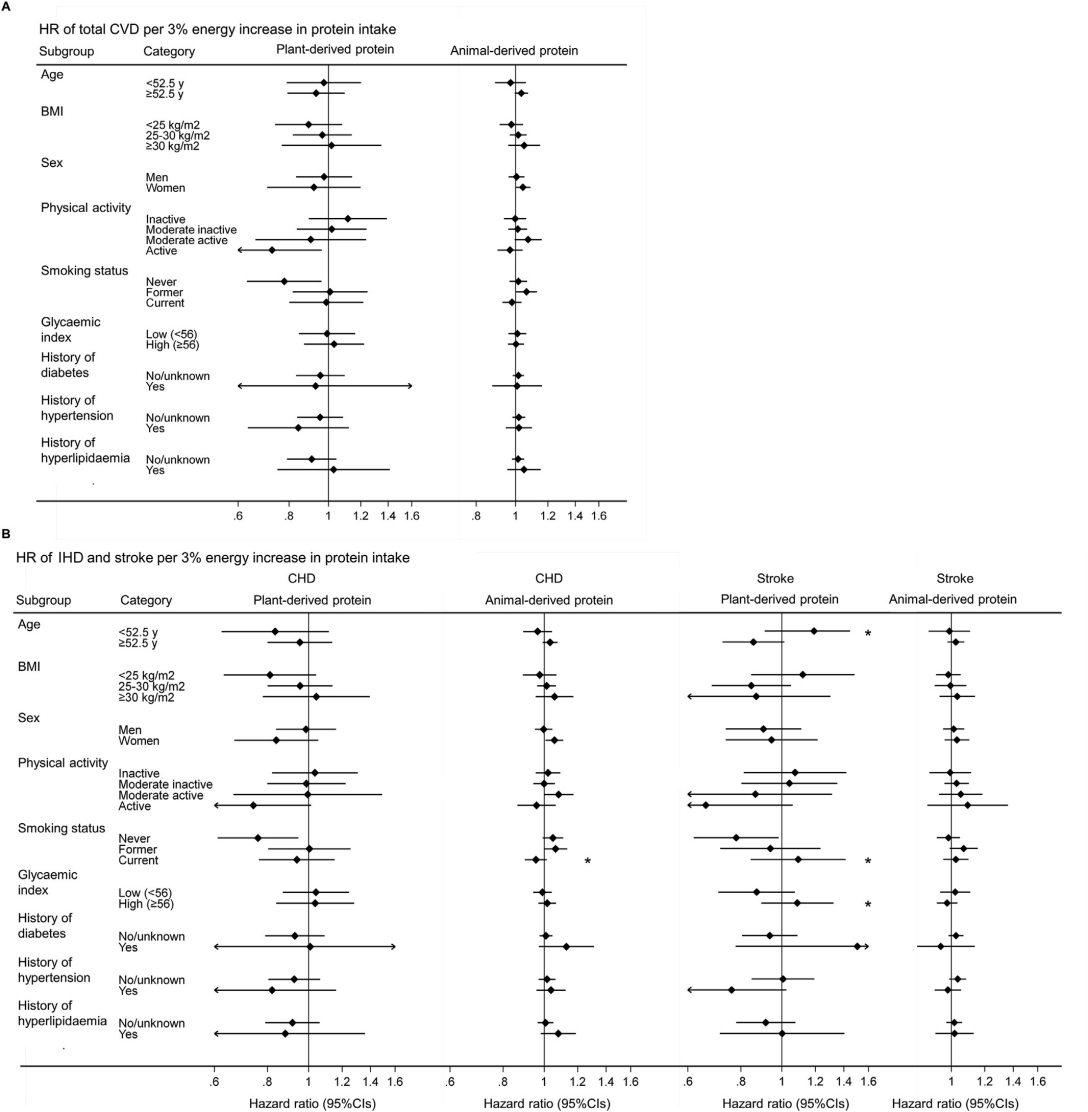
Stratified analysis of prospective association with cardiovascular diseases (CVDs) per 3% higher energy from dietary plant- or animal-derived protein intake. Proportional hazards models were used to estimate multivariable-adjusted hazard ratios (HRs) and 95% confidence intervals (95% CIs) for each 3% higher energy intake from plant- or animal-derived protein within each country separately, with age as the underlying time variable, and country-specific HRs (95% CIs) were combined in random-effects meta-analysis to obtain pooled effect estimates and 95% CIs. (A) Total CVD, (B) ischemic heart disease (IHD) and stroke. ∗Indicates *P* < 0.05 for the interaction analyses. The *P-*interaction with plant-derived protein on stroke was 0.02 for age, 0.004 for current smoker (compared with never) and 0.002 for glycemic index; *P-*interaction with animal-derived protein on IHD was 0.026 for current smoker (compared with never).

In the base model (analyses without macronutrient-specific isocaloric substitutions), for stroke, there was a significant interaction between plant-derived protein intake and each of baseline age (*β* = −0.007, *P*-interaction = 0.02), smoking status (*β* = 0.139, *P-*interaction = 0.004), and glycemic index (*β* = 0.014, *P-*interaction = 0.002), whereas for IHD an interaction was observed by smoking status (with animal-derived protein) (Figure 1). Although none of the above interaction analyses could pass the Bonferroni correction, we treated them as exploratory analyses and further conducted stratified analysis. In the stratified analysis, 3%TEI higher plant-derived protein intake was associated with 22% lower incidence of stroke (HR 0.78, 95% CI: 0.62, 0.99) among never smokers (Figure 1), but was not associated among former smokers (HR 0.94, 95% CI: 0.72, 1.24) or current smokers (HR 1.08, 95% CI: 0.83, 1.40). For the other factors (i.e., age, glycemic index) showing interaction with dietary protein intakes, we did not find significant associations in any of the stratified analyses.

### Associations of dietary protein intake with endpoint subtypes (fatal and nonfatal CVD, IHD, and stroke)

In the multivariable-adjusted models with and without specified substitutions, dietary plant- or animal-derived protein intake per 3%TEI was not associated with any of the CVD subtypes (Supplemental Tables 7 and 8). However, 3%TEI higher plant-derived protein to replace energy from red meat protein, processed meat protein, and dairy protein was associated with 48% (HR 0.52 (0.31, 0.88)), 61% (HR 0.39 (0.17, 0.90)), and 46% (HR 0.54 (0.30, 0.98)) lower incidence of fatal stroke, respectively (Table 3). None of the above analyses could pass the multiple testing correction.

### Dietary protein intake and blood lipids

**FIGURE 2:**
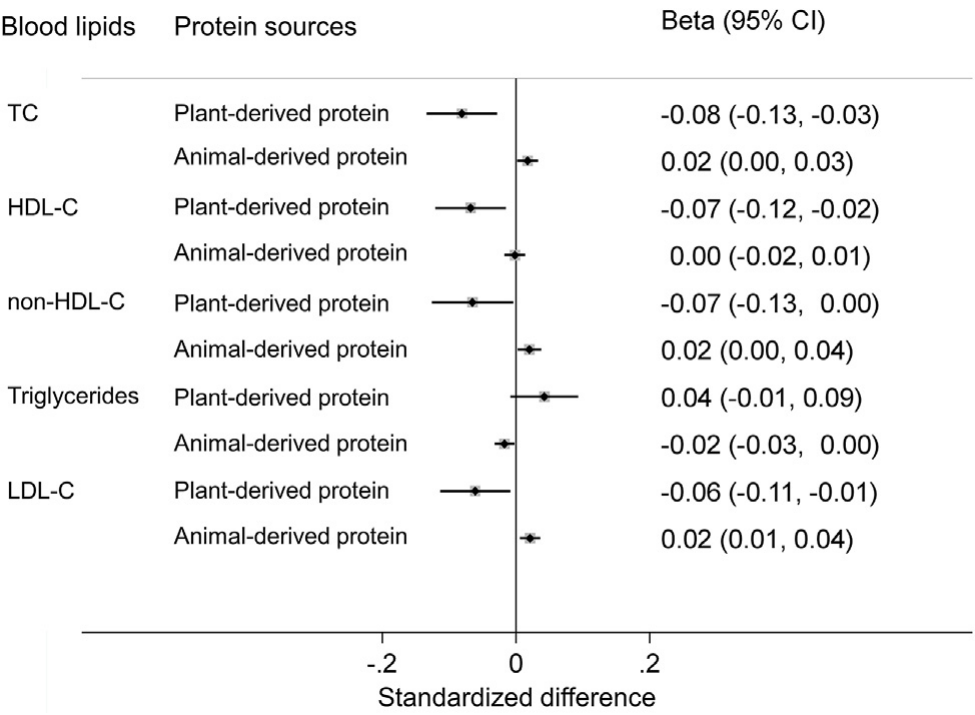
Association of plant- or animal-derived protein intake with blood lipids in the subcohort of EPIC-CVD study. Beta coefficients (95% confidence intervals) represented the standardized difference in the blood lipids (in standard deviation unit) per 3% total energy intake of plant-derived protein or animal-derived protein. Estimates were based on the multivariable-adjusted linear regression model, with adjustment for gender, age, study center, energy intake, education, physical activity, smoking status, alcohol, dietary fiber, glycemic index, BMI, self-reported history of diabetes, hypertension, and dyslipidemia. Triglycerides were log-transformed. The standard deviation was 1.13 mmol/L for TC, 0.42 mmol/L for HDL-C, 1.09 mmol/L for LDL-C, and 0.54 for log-transformed triglycerides. TC, total cholesterol; HDL-C, high-density lipoprotein-cholesterol; LDL-C, low-density lipoprotein-cholesterol.

In the cross-sectional analyses among the EPIC-CVD subcohort, higher plant-derived protein intake was associated with lower concentrations of TC, HDL-C, non-HDL-C, and LDL-C, whereas animal-derived protein intake was positively associated with TC, non-HDL-C, and LDL-C (Figure 2).

## Discussion

In this large prospective study involving participants from 7 European countries, there was no evidence for an association between plant- or animal-derived dietary protein intake and incident CVD, IHD, or stroke, in adjusted models without or with specified macronutrient substitutions. This was the case when replacing energy from each type of protein for the other type (e.g., when replacing animal- with plant-source protein), or from any of the other macronutrients, including carbohydrates, SFA, MUFA, or PUFA. However, there was suggestive evidence for smoking status being an effect modifier, with results suggesting that plant-derived protein intake was inversely associated with stroke among never smokers but not among current or former smokers. Moreover, plant-derived protein intake was inversely associated with fatal stroke when it replaced energy from red meat, processed meat, or dairy protein but not from poultry, fish, or egg sources of protein.

Prior evidence on associations between dietary protein intake and stroke risk has been inconsistent and inconclusive. No association was reported between total protein intake, plant or animal protein intake, and risk of total stroke or its subtypes, in modeled isocaloric substitutions for carbohydrate intake, in a cohort of men in the United States [16]. Similarly, plant protein intake was not associated with stroke risk in a cohort of females in the United States but animal protein intake was inversely associated [34]. In a Japanese study there was an inverse association between animal protein intake and cerebrovascular death, however, the association disappeared after adjusting for other nutrients (animal fat and cholesterol) [35], which was consistent with our null association in substitution analysis. In another recent Japanese cohort study with 19 y of follow-up, there was a 40% lower risk of stroke [36], comparing extreme quartiles (high vs. low) of plant protein intake. The PURE study in 18 countries reported null association of total protein intake with CVD risk with and without isocaloric substitution for carbohydrate intake, but no results were reported for plant or animal protein with CVD outcomes [7]. It is important to note that the majority of these prior studies did not examine the isocaloric substitution of dietary plant or animal protein for specific other macronutrients. Moreover, none of these studies examined outcomes of fatal compared with nonfatal stroke or examined the substitution of protein from specific dietary sources or food groups. Our present study included analyses on isocaloric macronutrient substitution models both for macronutrients and for specific protein-rich food groups and suggested that consideration of plant protein specifically replacing protein from different animal sources would be important in examining the association with stroke risk. Specifically, we observed that replacing protein from red or processed meat and dairy with plant-derived protein was associated with a lower risk of fatal stroke. This was further supported by our findings that plant-derived protein was associated with lower blood lipid levels.

To the best of our knowledge, our finding that the inverse association between plant protein and stroke risk was only observed among nonsmokers was not found or tested previously [16,[34], [35], [36], [37]]. This finding suggests that, assuming a causal association, smoking might attenuate a potential “protective association” of plant protein with stroke risk. This phenomenon was previously reported for an interaction between smoking and blood carotenoids (reflecting plant food intake) on the incidence of diabetes and insulin resistance [38]. The authors hypothesized that antioxidant metabolism and the oxidative defence system behave differently in smokers than in nonsmokers. This may similarly apply to the associations with plant-derived proteins that we observed for stroke, whether by consumption of antioxidants from plant protein–rich foods, by concomitant lower heme-iron–induced oxidative stress from animal-based foods, or by the specific amino acid composition of foods per se, for which the potential role in affecting oxidative stress remains largely unclear [39]. However, the possibility of a false-positive finding cannot be excluded in the context of the various analyses conducted in our investigation. Moreover, participants consuming higher amounts of plant-derived protein and lower amounts of animal-derived protein were more likely to be never smokers, which may have led to a greater contrast between plant-derived and animal-derived protein intake levels among this population subgroup—potentially increasing the ability to detect an association in this subgroup. This is similar for the observation that the association of plant-derived protein with total CVD was only significant among the active physical activity group. Nonetheless, our findings raise an important point and should be further investigated and replicated in diverse populations with varying macronutrient intakes and dietary patterns.

For IHD as endpoint, our null findings for animal protein and total IHD were generally consistent with several large studies among males and females in the United States [9,10,17,22]. In the present study we did not observe an interaction between animal protein intake and disease history (diabetes, hypertension, or hyperlipidaemia) or glycemic index for IHD risk, in contrast to a prospective cohort of men in the United States, where animal protein intake was not associated with risk of total, fatal, or nonfatal IHD, but there was evidence of interaction, with a positive association of animal protein with IHD among those without hypertension or with lower dietary glycemic index [10]. The lack of association of plant protein with total IHD in the present study is also in line with previous reports from several cohorts in the United States [9,10,17]. However, plant protein was inversely associated with fatal IHD, but not nonfatal IHD in several previous studies [8,10,17]. In addition, in the Iowa Women’s Health Study in the United States, plant protein was associated with a lower risk of total IHD, when modeled to replace animal protein or carbohydrates [22]. Sources of the differences in results between our study based in European countries and the previous United States studies are unclear. Further research should investigate the contribution of differences in dietary habits and sources of protein between countries, and also consider other, nondietary, factors explaining differences between studies within Europe and elsewhere.

Our current study provides evidence that dietary plant or animal protein intake was not associated with total CVD incidence in European populations. In contrast, prior evidence for the association of plant or animal protein intake with total CVD incidence is lacking in the published literature, with reports limited to total mortality or CVD mortality [8,14,40,41]. In 1 study, plant protein was not associated, and animal protein positively associated with CVD mortality among the 81,337 males and females from the Adventist Health Study-2 [40]. In another study, animal protein intake was positively associated with CVD mortality among 131,342 United States males and females, and plant protein intake was inversely associated but only in the presence of ≥1 unhealthy lifestyle factor including smoking, heavy alcohol intake, overweight or obesity, and physical inactivity [8].

There are several strengths of the current study. This is a large prospective epidemiological study of plant- and animal-derived protein intake and CVD incidence across European populations, and the only one to apply standardized methods across a range of European countries. Its inclusion of diverse populations extending across South to North Europe increased dietary heterogeneity and enabled the investigation of a diverse range in intake levels and food sources. Among limitations, our study is observational and cannot establish a cause and effect inference. We could employ only a single assessment of habitual dietary intake at baseline, with the inability to evaluate the effect of potential changes in diet during follow-up. The analyses included many statistical tests and the results could not pass the multiple testing correction. Therefore, the analyses and results should be considered as exploratory and should be tested in further research. Furthermore, despite our adjustment for a range of potential confounding factors, residual confounding due to unmeasured or imprecisely measured factors may have biased our findings, such as trans- fat intake. Nevertheless, trans- fat intake across Western Europe (∼1%TEI) has been lower than in North America (∼4%TEI) [42], making our results less likely to be materially changed by the residual confounding from trans- fats. Finally, this study is based on white individuals in Europe and therefore generalizability to other populations is limited.

Although our current study focused on protein intake, it is relevant for public health messages to acknowledge the wider context that protein-rich foods, rather than protein as a nutrient itself, may be more important for the prevention of CVD. Therefore, the potential mechanisms linking plant protein and cardiovascular health may involve the healthy food matrix of plant protein, with plant protein–rich foods being rich in fiber, polyphenols, and phytochemicals. Meanwhile, amino acids predominant in plant protein, such as glutamic acid, may also benefit cardiovascular health [43]. The importance of considering foods (rather than individual nutrients alone) is also highlighted by our finding that plant-derived protein intake was inversely associated with CVD, IHD, and stroke in additional analyses not adjusting for dietary fiber intake. Because plant protein and fiber tend to be highly correlated [44], this finding suggests that in practice, the potential health effects of consuming protein from plant foods – which also contain other healthy nutrients such as fiber – on CVD risk may be greater than the health effects expected from observed associations of plant protein alone with CVD risk.

In conclusion, overall, there was no association between plant-derived or animal-derived protein intake with total CVD or with IHD or stroke incidence but the replacement of protein from red meat, processed meat or dairy with plant-derived protein might be associated with a lower risk of fatal stroke. An inverse association between plant-derived protein and stroke was found among never smokers but not ever smokers, suggesting that a beneficial association may be blunted in smokers. These findings suggest that plant-derived protein intake may have a potential beneficial role for the prevention of stroke. Accounting also for other behaviors such as smoking and food groups to consume together or avoid, further research on protein sources is warranted in diverse populations.

## Acknowledgments

We thank all EPIC study participants and staff for their contribution to the study. We acknowledge the laboratory teams at the Medical Research Council (MRC) Epidemiology Unit for sample management and Cambridge Genomic Services for genotyping, Sarah Spackman (for EPIC-CVD) and Nicola Kerrison (EPIC-InterAct Data Manager, MRC Epidemiology Unit) for data management, and the team at the EPIC-CVD Coordinating Centre for study coordination and administration.

## Author contributions

The authors’ responsibilities were as follows – JD, ASB: co-ordinated the EPIC-CVD project; MS, ASB, NGF: conceived and designed the current study; MS, J-SZ: analyzed the data; J-SZ, MS, NGF: drafted the manuscript; J-SZ, MS, FI, HF, LJ, IS, MS, EW, ASB, NGF: edited the manuscript with input from the working group; NGF, ASB: guarantors of this work and had full access to all the data in the study and took responsibility for the integrity of the data and the accuracy of the data analysis; and all other authors revised the manuscript critically for intellectual content, gave final approval of the version to be published, and they have contributed to the manuscript and interpretation of data; and all authors: approved the final version of the manuscript.

## Conflict of interest

ASB reports institutional grants from AstraZeneca, Bayer, Biogen, BioMarin, Bioverativ, Novartis, Regeneron, and Sanofi. The other authors declare no conflict of interest.

## Funding

This work was supported by core funding from the British Heart Foundation (RG/13/13/30194; RG/18/13/33946), BHF Cambridge CRE (RE/18/1/34212), NIHR Cambridge Biomedical Research Centre (BRC-1215-20014; NIHR203312) [∗], European Research Council (268834), European Commission Framework Programme 7 (HEALTH-F2-2012-279233) and the EU FP6 Programme (LSHM_CT_2006_037197 for EPIC-InterAct). The coordination of EPIC is financially supported by International Agency for Research on Cancer (IARC) and also by the Department of Epidemiology and Biostatistics, School of Public Health, Imperial College London, which has additional infrastructure support provided by the NIHR Imperial Biomedical Research Centre (BRC). The national cohorts are supported by Danish Cancer Society (Denmark); Ligue Contre le Cancer, Institut Gustave Roussy, Mutuelle Générale de l’Education Nationale, Institut National de la Santé et de la Recherche Médicale (INSERM) (France); German Cancer Aid, German Cancer Research Center (DKFZ), German Institute of Human Nutrition PotsdamRehbruecke (DIfE), Federal Ministry of Education and Research (BMBF) (Germany); Associazione Italiana per la Ricerca sul Cancro-AIRC-Italy, Compagnia di SanPaolo and National Research Council (Italy); Dutch Ministry of Public Health, Welfare and Sports (VWS), Netherlands Cancer Registry (NKR), Dutch Prevention Funds, Dutch ZON (Zorg Onderzoek Nederland), World Cancer Research Fund (WCRF), Statistics Netherlands (The Netherlands); Health Research Fund (FIS) – Instituto de Salud Carlos III (ISCIII), Regional Governments of Andalucía, Asturias, Basque Country, Murcia and Navarra, and the Catalan Institute of Oncology – ICO (Spain); Swedish Cancer Society, Swedish Research Council and County Councils of Skåne and Västerbotten (Sweden); Cancer Research UK (14136 to EPIC-Norfolk; C8221/A29017 to EPIC-Oxford), Medical Research Council (1000143 to EPIC-Norfolk; MR/M012190/1 to EPIC-Oxford). (United Kingdom). NJW, MS, FI, JSZ, and NGF acknowledge support from the MRC Epidemiology Unit (MC_UU_00006/1 and MC_UU_00006/3). NJW and NGF acknowledge support from NIHR∗ Cambridge BRC: Nutrition, Diet, and Lifestyle Research Theme (IS-BRC-1215-20014), and Nutrition, Obesity, Metabolism and Endocrinology Theme (NIHR203312), and NGF is an NIHR Senior Investigator Award holder (NIHR202397). MS was funded by the Alpro Foundation while based at the Cardiovascular Epidemiology Unit (CEU) over 01/2015 to 08/2016. DBI acknowledges support from the Independent Research Fund Denmark (1057-00016B). JSZ received funding from the European Union’s Horizon 2020 research and innovation program under the Marie Sklodowska-Curie grant agreement No 701708. JD holds a BHF Professorship and a NIHR Senior Investigator Award [∗]. ∗The views expressed are those of the author(s) and not necessarily those of the NIHR or the Department of Health and Social Care. For open access, the author has applied a Creative Commons Attribution (CC, BY) licence to any Author Accepted Manuscript version arising from this submission.

## Data availability

This study used EPIC data provided by EPIC centers. Details on how to access EPIC data and biospecimens are available at: https://epic.iarc.fr/access/index.php.

## Disclaimer

Where authors are identified as personnel of the International Agency for Research on Cancer/World Health Organization, the authors alone are responsible for the views expressed in this article and they do not necessarily represent the decisions, policy, or views of the International Agency for Research on Cancer/World Health Organization.
