# Supplementary material for: Dietary intake of plant- and animal-derived protein and incident cardiovascular diseases: the pan-European EPIC-CVD case–cohort study
Source: Am J Clin Nutr. 2024 Mar 11;119(5):1164–74. doi: 10.1016/j.ajcnut.2024.03.006 (PMC11130694; doi:10.1016/j.ajcnut.2024.03.006)
Supplement: Multimedia component 1 [file mmc1.docx]

**Online Supplemental File**

**“Dietary intake of plant- and animal-derived protein and incident cardiovascular diseases: the pan-European EPIC-CVD case-cohort study”**

Ju-Sheng Zheng et al.

***Supplemental Tables***

**Supplemental Table 1 Contribution of food groups to plant or animal-derived protein in each country in the subcohort of EPIC-CVD study**

**Supplemental Table 2 Pairwise correlation of dietary macronutrient intake in the EPIC-CVD study**

**Supplemental Table 3 Sensitivity analysis for the prospective association between dietary protein intake and cardiovascular disease, coronary heart disease and stroke: EPIC-CVD Study**

**Supplemental Table 4 Hazard ratios (95% CIs) of cardiovascular disease, coronary heart disease and stroke by quintiles of dietary plant- or animal-derived protein to replace energy from other macronutrients: EPIC-CVD Study**

**Supplemental Table 5 Hazard ratios (95% CIs) of cardiovascular disease, coronary heart disease and stroke per 3% total energy intake of protein from different animal food groups***

**Supplemental Table 6 Hazard ratios (95% CIs) of cardiovascular disease, coronary heart disease and stroke by quintiles of total protein intake: EPIC-CVD Study**

**Supplemental Table 7 Prospective association between dietary plant or animal-derived protein and subtypes of cardiovascular disease: EPIC-CVD Study**

**Supplemental Table 8 Prospective association of dietary protein and subtypes of cardiovascular disease in isocaloric substitution analysis replacing 3% higher energy intake from plant, animal or total protein with energy from other sources: EPIC-CVD Study**

***Supplemental Figures***

**Supplemental Figure 1. Study flowchart**

**Supplemental Figure 2. Distribution of observed dietary protein intake in the subcohort: EPIC-CVD Study**

**Supplemental Figure 3 Prospective association between dietary protein and total cardiovascular disease, coronary heart disease and stroke stratified by country: EPIC-CVD Study**

**Supplemental Figure 4 Prospective association between protein consumption and cardiovascular diseases in isocaloric substitution analyses replacing 3% energy intake from different macronutrients with plant- or animal-derived protein: EPIC-CVD Study**

**Supplemental Figure 5 Hazard ratios (95%CIs) of total cardiovascular disease, coronary heart disease and stroke for each 3% higher energy intake from plant- derived protein to replace energy from other macronutrients stratified by country: EPIC-CVD Study**

**Supplemental Figure 6 Hazard ratios (95%CIs) of total cardiovascular disease, coronary heart disease and stroke for each 3% higher energy intake from animal- derived protein to replace energy from other macronutrients stratified by country: EPIC-CVD Study**

**Supplemental Figure 7 Hazard ratios (95%CIs) of total cardiovascular disease, coronary heart disease and stroke for each 3% higher energy intake from total protein to replace energy from other macronutrients stratified by countries: EPIC-CVD Study**

**Supplemental Table 1 Contribution of food groups to plant or animal-derived protein in each country in the subcohort of EPIC-CVD study**

|  | Total subcohort (n=15141) | Spain (n=3674) | Italy (n=2006) | UK  (n=1102) | Netherlands (1357) | Germany (2031) | Denmark  (2057) | Sweden (n=2914) |
| --- | --- | --- | --- | --- | --- | --- | --- | --- |
| **Plant-derived protein^1^** |  |  |  |  |  |  |  |  |
| Plant-derived protein, g/d | 26.8 (10.7) | 31.1 (12) | 31.4 (12.8) | 27.1 (10) | 24.8 (7.8) | 22.1 (7.6) | 27.7 (7.9) | 21.7 (8.3) |
| Plant-derived protein, %TEI | 5.4 (1.4) | 6.2 (1.4) | 5.7 (1.4) | 5.9 (2.0) | 5.5 (1.1) | 4.8 (1.2) | 5.5 (1.0) | 4.4 (1.1) |
| Potatoes/tubers, % | 8.0 (6.3) | 4.8 (3.2) | 2.1 (1.9) | 9.9 (6.2) | 9.2 (5.6) | 8.5 (4.6) | 12.8 (6.7) | 11.2 (7.0) |
| Vegetables, % | 12.3 (7.7) | 13.3 (8.8) | 12.3 (6.8) | 21.6 (9.0) | 10.2 (4.7) | 11.1 (5.3) | 13.2 (6.3) | 8.6 (6.2) |
| Legumes, % | 6.1 (7.6) | 14.5 (7.8) | 3.6 (5.1) | 4.6 (3.6) | 2.9 (2.6) | 1.9 (2.4) | 0.4 (0.6) | 3.9 (6.4) |
| Fruits/nuts/seeds, % | 8.9 (7.2) | 11.0 (8.8) | 7.8 (4.8) | 10.1 (7.6) | 12.9 (8.3) | 7.9 (5.9) | 6.2 (4.9) | 7.3 (6.0) |
| Cereal and cereal products, % | 55.1 (14.4) | 49.0 (13.8) | 68.5 (12.3) | 45.3 (13.7) | 54.0 (11.0) | 54.4 (12.4) | 53.2 (11.3) | 59.8 (13.2) |
| Other plant sources, %^2^ | 11 (7.7) | 8.6 (6.7) | 6.3 (3.8) | 9.2 (7.0) | 11.1 (5.9) | 16.4 (9.7) | 14.4 (6.9) | 11.7 (7.6) |
|  |  |  |  |  |  |  |  |  |
| **Animal-derived protein*** |  |  |  |  |  |  |  |  |
| Animal-derived protein, g/d | 56.5 (24) | 68.6 (25.6) | 58.6 (22.3) | 48.0 (19.8) | 51.7 (18.0) | 39.9 (16.9) | 65.5 (24.4) | 50.6 (19.9) |
| Animal-derived protein, %TEI | 11.5 (3.7) | 13.8 (3.7) | 10.9 (3.2) | 10.4 (4.1) | 11.6 (3.3) | 8.6 (2.8) | 13.0 (3.0) | 10.3 (2.8) |
| Dairy and dairy products, % | 33.3 (17.5) | 24.2 (15.0) | 34.6 (14.2) | 37.4 (19.5) | 42.4 (17.3) | 29.9 (16.2) | 29.3 (15) | 43.2 (16.3) |
| Red meat, % | 22.7 (13.5) | 17.9 (11.2) | 24.3 (11.9) | 21.5 (13.7) | 33.6 (15) | 20.4 (11.6) | 33.6 (11.7) | 16.4 (10.4) |
| Poultry, % | 11.8 (9.1) | 15.8 (10) | 13.6 (8.8) | 17.1 (10.4) | 7.1 (6.8) | 9.4 (7.7) | 10.1 (7.4) | 7.8 (6.1) |
| Processed meat, % | 12.1 (9.7) | 11.0 (8.9) | 9.4 (6.5) | 10.2 (7.3) | 9 .0 (7.7) | 24 (12.2) | 6.9 (4.4) | 12.8 (7.8) |
| Fish/shellfish, % | 12.6 (10.2) | 20.5 (11.4) | 11.2 (7.8) | 13.0 (10.6) | 3.2 (3.5) | 9.0 (7.1) | 12.8 (6.4) | 10.1 (9.2) |
| Egg and egg products, % | 4.7 (4.0) | 5.7 (4.1) | 4.3 (2.9) | 4.3 (5.4) | 4.6 (3.4) | 5.4 (4.5) | 4.9 (3.9) | 3.3 (3.6) |
| Other animal sources, %^3^ | 5.1 (6.6) | 7.6 (8.3) | 3.2 (2.6) | 2.1 (4.3) | 1.2 (1.5) | 2.2 (2.2) | 2.7 (2.3) | 10.0 (7.9) |

1 The contributions of food groups to plant protein or to animal protein were presented as % contribution.

2 Other plant sources included non-alcoholic beverages, cakes and biscuits, sugar and confectionary, condiments and sauces, soups, snacks and others.

3 Other animal sources included offals, game, condiments and sauces, soups, bouillon, snacks and others.

**Supplemental Table 2 Pairwise correlation of dietary macronutrient intake in the EPIC-CVD study**^1^

|  | Mean Spearman correlation coefficients (minimum, maximum) across seven countries ^2^ | | | | | | |
| --- | --- | --- | --- | --- | --- | --- | --- |
|  | Total protein | Plant-derived protein | Animal-derived protein | Carbohydrate | SFA | MUFA | PUFA |
| Total protein | 1 |  |  |  |  |  |  |
| Plant-derived protein | 0.02 (-0.21, 0.18) | 1 |  |  |  |  |  |
| Animal-derived protein | 0.91 (0.88, 0.94) | -0.26 (-0.48, -0.04) | 1 |  |  |  |  |
| Carbohydrate | -0.43 (-0.60, -0.26) | 0.38 (0.21, 0.58) | -0.55 (-0.75, -0.45) | 1 |  |  |  |
| SFA | -0.04 (-0.19, 0.31) | -0.53 (-0.72, -0.39) | 0.16 (0.02, 0.50) | -0.68 (-0.78, -0.51) | 1 |  |  |
| MUFA | 0.03 (-0.14, 0.19) | -0.35 (-0.48, -0.17) | 0.17 (0.03, 0.36) | -0.79 (-0.88, -0.65) | 0.58 (0.25, 0.72) | 1 |  |
| PUFA | 0.03 (-0.23, 0.19) | 0.01 (-0.19, 0.16) | 0.03 (-0.24, 0.23) | -0.41 (-0.57, -0.33) | 0.02 (-0.08, 0.21) | 0.34 (-0.03, 0.65) | 1 |

1 EPIC-CVD subcohort participants was used to calculate the statistics across the seven countries (n=15141).

2 Pairwise Spearman correlation coefficients were calculated in each country. The average, minimum, and maximum are presented.

**Supplemental Table 3 Sensitivity analysis for the prospective association between dietary protein intake and cardiovascular disease, coronary heart disease and stroke: EPIC-CVD Study**

|  | Model | Total N | Plant-derived protein | Animal-derived protein | Total protein |
| --- | --- | --- | --- | --- | --- |
| CVD |  |  |  |  |  |
|  | HR, multivariable-adjusted^1^ | 30519 | 0.95 (0.84, 1.09) | 1.02 (0.99, 1.05) | 1.02 (0.99, 1.06) |
|  | HR, multivariable-adjusted, including height as an additional covariate | 30519 | 0.95 (0.84, 1.08) | 1.02 (0.99, 1.05) | 1.02 (0.99, 1.06) |
|  | HR, multivariable-adjusted, excluding glycaemic index as a covariate | 20519 | 1.06 (0.96, 1.16) | 1.00 (0.97, 1.03) | 1.01 (0.98, 1.05) |
|  | HR, multivariable-adjusted, excluding fibre intake as a covariate | 30519 | 0.87 (0.77, 0.98) | 1.03 (1.00, 1.06) | 1.02 (0.99, 1.06) |
|  | HR, multivariable-adjusted, excluding participants with extreme energy intake^2^ | 29903 | 0.96 (0.85, 1.09) | 1.01 (0.98, 1.05) | 1.02 (0.99, 1.06) |
|  | HR, multivariable-adjusted, excluding incident cases within first 2 years’ follow-up | 28870 | 0.94 (0.82, 1.07) | 1.01 (0.98, 1.04) | 1.02 (0.98, 1.05) |
|  | HR, multivariable-adjusted, excluding participants with self-reported history of diabetes or cancer | 28089 | 0.94 (0.81, 1.08) | 1.02 (0.98, 1.05) | 1.02 (0.99, 1.06) |
|  | HR, multivariable-adjusted, excluding participants using lipid-lowing drugs or with history of high cholesterol | 25485 | 0.91 (0.79, 1.04) | 1.01 (0.98, 1.05) | 1.02 (0.97, 1.07) |
|  | HR, multivariable-adjusted, using original total energy variable, including alcohol intake^3^ | 30519 | 0.93 (0.81, 1.07) | 1.02 (0.98, 1.05) | 1.02 (0.99, 1.06) |
|  | HR, multivariable-adjusted, imputing the missing covariates | 32017 | 0.94 (0.81, 1.09) | 1.03 (0.99, 1.07) | 1.04 (1.00, 1.08) |
|  | HR, multivariable-adjusted, including blood lipids (HDL-C, non-HDL-C, triglycerides) as additional covariates | 27009 | 0.93 (0.80, 1.07) | 1.02 (0.99, 1.05) | 1.03 (0.99, 1.07) |
| CHD |  |  |  |  |  |
|  | HR, multivariable-adjusted^1^ | 25346 | 0.95 (0.83, 1.09) | 1.01 (0.98, 1.05) | 1.02 (0.98, 1.06) |
|  | HR, multivariable-adjusted, including height as an additional covariate | 25346 | 0.95 (0.83, 1.09) | 1.01 (0.98, 1.05) | 1.02 (0.98, 1.06) |
|  | HR, multivariable-adjusted, excluding glycaemic index as a covariate | 25346 | 1.04 (0.93, 1.15) | 1.00 (0.97, 1.04) | 1.01 (0.98, 1.05) |
|  | HR, multivariable-adjusted, excluding fibre intake as a covariate | 25346 | 0.88 (0.78, 0.99) | 1.03 (0.99, 1.06) | 1.02 (0.98, 1.06) |
|  | HR, multivariable-adjusted, excluding participants with extreme energy intake^2^ | 24825 | 0.96 (0.84, 1.09) | 1.01 (0.98, 1.05) | 1.02 (0.98, 1.06) |
|  | HR, multivariable-adjusted, excluding incident cases within first 2 years’ follow-up | 24218 | 0.94 (0.81, 1.10) | 1.01 (0.98, 1.05) | 1.02 (0.98, 1.06) |
|  | HR, multivariable-adjusted, excluding participants with self-reported history of diabetes or cancer | 23409 | 0.95 (0.81, 1.10) | 1.01 (0.97, 1.05) | 1.01 (0.97, 1.06) |
|  | HR, multivariable-adjusted, excluding participants using lipid-lowing drugs or with history of high cholesterol | 21030 | 0.92 (0.78, 1.07) | 1.01 (0.96, 1.05) | 1.01 (0.95, 1.07) |
|  | HR, multivariable-adjusted, using original total energy variable, including alcohol intake^3^ | 25346 | 0.94 (0.81, 1.09) | 1.02 (0.99, 1.06) | 1.03 (0.99, 1.07) |
|  | HR, multivariable-adjusted, imputing the missing covariates | 26654 | 0.93 (0.80, 1.08) | 1.03 (0.99, 1.07) | 1.03 (0.99, 1.08) |
|  | HR, multivariable-adjusted, including blood lipids (HDL-C, non-HDL-C, triglycerides) as additional covariates | 23515 | 0.92 (0.81, 1.06) | 1.02 (0.98, 1.06) | 1.03 (0.99, 1.07) |
| Stroke |  |  |  |  |  |
|  | HR, multivariable-adjusted^1^ | 21213 | 0.94 (0.81, 1.08) | 1.02 (0.98, 1.06) | 1.03 (0.98, 1.07) |
|  | HR, multivariable-adjusted, including height as an additional covariate | 21213 | 0.94 (0.81, 1.08) | 1.01 (0.98, 1.05) | 1.02 (0.98, 1.07) |
|  | HR, multivariable-adjusted, excluding glycaemic index as a covariate | 21213 | 1.07 (0.95, 1.21) | 0.99 (0.95, 1.03) | 1.00 (0.96, 1.05) |
|  | HR, multivariable-adjusted, excluding fibre intake as a covariate | 21213 | 0.83 (0.73, 0.93) | 1.03 (0.99, 1.08) | 1.02 (0.98, 1.07) |
|  | HR, multivariable-adjusted, excluding participants with extreme energy intake^2^ | 20802 | 0.94 (0.82, 1.09) | 1.01 (0.97, 1.05) | 1.02 (0.98, 1.07) |
|  | HR, multivariable-adjusted, excluding incident cases within first 2 years’ follow-up | 20612 | 0.92 (0.79, 1.07) | 1.00 (0.96, 1.04) | 1.01 (0.97, 1.05) |
|  | HR, multivariable-adjusted, excluding participants with self-reported history of diabetes or cancer | 19725 | 0.93 (0.80, 1.09) | 1.02 (0.98, 1.06) | 1.03 (0.97, 1.08) |
|  | HR, multivariable-adjusted, excluding participants using lipid-lowing drugs or with history of high cholesterol | 18086 | 0.92 (0.78, 1.08) | 1.01 (0.97, 1.06) | 1.02 (0.96, 1.08) |
|  | HR, multivariable-adjusted, using original total energy variable, including alcohol intake^3^ | 21213 | 0.90 (0.77, 1.05) | 1.01 (0.96, 1.05) | 1.01 (0.96, 1.06) |
|  | HR, multivariable-adjusted, imputing the missing covariates | 21916 | 0.94 (0.82, 1.09) | 1.02 (0.98, 1.06) | 1.03 (0.99, 1.08) |
|  | HR, multivariable-adjusted, including blood lipids (HDL-C, non-HDL-C, triglycerides) as additional covariates | 18610 | 0.90 (0.76, 1.06) | 1.01 (0.97, 1.06) | 1.03 (0.97, 1.08) |

1 Proportional hazards models were used to estimate multivariable-adjusted hazard ratios (HRs) and 95% confidence intervals (95% CIs) per 3% energy increase of protein intake within each country separately, with age as the underlying time variable. Country-specific HRs (95% CIs) were combined in random-effects meta-analysis to obtain pooled effect estimates and 95% CIs. Covariates included in the multivariable-adjusted model were for age (years), sex (men, women), centre, energy intake (kcal/day), education (low, medium, high), physical activity (inactive, moderately inactive, moderately active, active), smoking (current, former, never), alcohol (0, 0-≤6, 6-≤12, 12-≤24, >24 grams/day), dietary fibre (continuous), glycaemic index (continuous), body-mass index (continuous), reported history of diabetes, hypertension and hyperlipidaemia. No specific replacement of energy from other macronutrients was performed in the models.

2 Implausible energy included total energy intake<500 or >3500 kcal/day for women, and <800 or >4000 kcal/day for men.

3 Dietary protein intake (% of total energy intake) was calculated based on conventional total energy intake, including carbohydrate, fat, protein and alcohol consumption.

**Supplemental Table 4 Hazard ratios (95% CIs) of cardiovascular disease, coronary heart disease, and stroke by quintiles of dietary plant- or animal-derived protein to replace energy from other macronutrients: EPIC-CVD Study**^1^

|  |  | Hazard ratios (95%CIs), multivariable adjusted^2^ | | | | |
| --- | --- | --- | --- | --- | --- | --- |
|  | Substitution sources | Q1 | Q2 | Q3 | Q4 | Q5 |
| CVD |  |  |  |  |  |  |
| Plant-derived protein | No substitution | 1.0 (ref) | 0.96 (0.84, 1.09) | 0.96 (0.86, 1.07) | 0.99 (0.8, 1.21) | 0.95 (0.79, 1.15) |
|  | SFA | 1.0 (ref) | 0.97 (0.85, 1.12) | 0.97 (0.86, 1.09) | 1.02 (0.82, 1.27) | 1.03 (0.82, 1.29) |
|  | MUFA | 1.0 (ref) | 0.96 (0.83, 1.10) | 0.94 (0.84, 1.06) | 0.98 (0.78, 1.24) | 0.97 (0.75, 1.24) |
|  | PUFA | 1.0 (ref) | 0.97 (0.85, 1.11) | 0.97 (0.86, 1.10) | 1.02 (0.83, 1.24) | 1.03 (0.84, 1.25) |
|  | Carbohydrate | 1.0 (ref) | 0.97 (0.85, 1.11) | 0.96 (0.85, 1.08) | 1.01 (0.81, 1.24) | 1.01 (0.80, 1.26) |
|  | Animal-derived protein | 1.0 (ref) | 0.96 (0.84, 1.10) | 0.95 (0.84, 1.07) | 0.99 (0.80, 1.22) | 0.98 (0.78, 1.22) |
| Animal-derived protein | No substitution | 1.0 (ref) | 0.92 (0.83, 1.01) | 0.93 (0.84, 1.03) | 0.89 (0.80, 1.00) | 1.06 (0.90, 1.24) |
|  | SFA | 1.0 (ref) | 0.91 (0.82, 1.02) | 0.92 (0.82, 1.04) | 0.89 (0.77, 1.02) | 1.08 (0.91, 1.29) |
|  | MUFA | 1.0 (ref) | 0.88 (0.79, 0.98) | 0.87 (0.76, 0.99) | 0.81 (0.69, 0.94) | 0.91 (0.72, 1.17) |
|  | PUFA | 1.0 (ref) | 0.89 (0.80, 1.00) | 0.89 (0.78, 1.01) | 0.85 (0.73, 0.99) | 0.98 (0.80, 1.20) |
|  | Carbohydrate | 1.0 (ref) | 0.92 (0.83, 1.01) | 0.92 (0.83, 1.03) | 0.88 (0.78, 0.99) | 1.06 (0.87, 1.30) |
|  | Plant-derived protein | 1.0 (ref) | 0.89 (0.79, 1.00) | 0.89 (0.75, 1.05) | 0.84 (0.71, 1.00) | 0.96 (0.74, 1.24) |
| CHD |  |  |  |  |  |  |
| Plant-derived protein | No substitution | 1.0 (ref) | 0.98 (0.83, 1.15) | 0.98 (0.85, 1.13) | 1.06 (0.84, 1.34) | 0.95 (0.74, 1.22) |
|  | SFA | 1.0 (ref) | 0.99 (0.83, 1.19) | 1.00 (0.84, 1.18) | 1.10 (0.86, 1.39) | 1.01 (0.75, 1.36) |
|  | MUFA | 1.0 (ref) | 0.98 (0.82, 1.18) | 0.98 (0.83, 1.15) | 1.06 (0.81, 1.40) | 0.97 (0.70, 1.34) |
|  | PUFA | 1.0 (ref) | 1.00 (0.84, 1.18) | 1.00 (0.86, 1.18) | 1.10 (0.88, 1.39) | 1.03 (0.78, 1.37) |
|  | Carbohydrate | 1.0 (ref) | 0.99 (0.83, 1.18) | 0.99 (0.85, 1.15) | 1.08 (0.85, 1.38) | 1.00 (0.74, 1.34) |
|  | Animal-derived protein | 1.0 (ref) | 0.98 (0.83, 1.17) | 0.98 (0.84, 1.15) | 1.07 (0.84, 1.36) | 0.97 (0.73, 1.30) |
| Animal-derived protein | No substitution | 1.0 (ref) | 0.94 (0.84, 1.05) | 0.95 (0.84, 1.06) | 0.91 (0.80, 1.03) | 1.08 (0.94, 1.25) |
|  | SFA | 1.0 (ref) | 0.93 (0.82, 1.05) | 0.92 (0.8, 1.07) | 0.89 (0.76, 1.05) | 1.08 (0.89, 1.32) |
|  | MUFA | 1.0 (ref) | 0.91 (0.80, 1.02) | 0.89 (0.77, 1.03) | 0.83 (0.70, 0.99) | 0.94 (0.73, 1.22) |
|  | PUFA | 1.0 (ref) | 0.92 (0.81, 1.04) | 0.90 (0.78, 1.04) | 0.87 (0.73, 1.03) | 1.02 (0.82, 1.28) |
|  | Carbohydrate | 1.0 (ref) | 0.95 (0.84, 1.06) | 0.94 (0.83, 1.06) | 0.90 (0.79, 1.03) | 1.09 (0.90, 1.32) |
|  | Plant-derived protein | 1.0 (ref) | 0.91 (0.80, 1.04) | 0.89 (0.73, 1.09) | 0.86 (0.69, 1.07) | 0.99 (0.76, 1.29) |
| Stroke |  |  |  |  |  |  |
| Plant-derived protein | No substitution | 1.0 (ref) | 0.93 (0.83, 1.05) | 0.95 (0.82, 1.08) | 0.91 (0.78, 1.08) | 0.95 (0.78, 1.17) |
|  | SFA | 1.0 (ref) | 0.94 (0.84, 1.06) | 0.95 (0.83, 1.10) | 0.94 (0.80, 1.12) | 1.04 (0.84, 1.29) |
|  | MUFA | 1.0 (ref) | 0.93 (0.83, 1.05) | 0.93 (0.80, 1.08) | 0.91 (0.76, 1.09) | 0.97 (0.77, 1.23) |
|  | PUFA | 1.0 (ref) | 0.93 (0.83, 1.06) | 0.94 (0.81, 1.09) | 0.92 (0.76, 1.10) | 0.98 (0.78, 1.24) |
|  | Carbohydrate | 1.0 (ref) | 0.94 (0.83, 1.06) | 0.94 (0.82, 1.09) | 0.92 (0.78, 1.10) | 1.00 (0.81, 1.25) |
|  | Animal-derived protein | 1.0 (ref) | 0.93 (0.82, 1.05) | 0.93 (0.81, 1.07) | 0.91 (0.77, 1.08) | 0.99 (0.79, 1.22) |
| Animal-derived protein | No substitution | 1.0 (ref) | 0.87 (0.77, 0.98) | 0.92 (0.81, 1.04) | 0.86 (0.75, 0.98) | 1.00 (0.79, 1.25) |
|  | SFA | 1.0 (ref) | 0.87 (0.77, 0.99) | 0.91 (0.78, 1.05) | 0.86 (0.73, 1.02) | 1.06 (0.83, 1.36) |
|  | MUFA | 1.0 (ref) | 0.85 (0.74, 0.97) | 0.87 (0.74, 1.02) | 0.79 (0.65, 0.96) | 0.92 (0.66, 1.28) |
|  | PUFA | 1.0 (ref) | 0.84 (0.73, 0.96) | 0.85 (0.73, 1.00) | 0.78 (0.65, 0.95) | 0.90 (0.62, 1.31) |
|  | Carbohydrate | 1.0 (ref) | 0.87 (0.77, 0.98) | 0.90 (0.79, 1.03) | 0.85 (0.74, 0.98) | 1.02 (0.79, 1.31) |
|  | Plant-derived protein | 1.0 (ref) | 0.86 (0.74, 0.99) | 0.88 (0.74, 1.05) | 0.82 (0.66, 1.01) | 0.99 (0.66, 1.47) |

1 Proportional hazards models were used to estimate hazard ratios (HRs) and 95% confidence intervals (95% CIs) for each quintile of plant- or animal- derived protein within each country separately, with age as the underlying time variable. Country-specific HRs (95% CIs) were combined in random-effects meta-analysis to obtain pooled effect estimates and 95% CIs.

2 The multivariable-adjusted HR included adjustment for age (years), sex (men, women), centre, energy intake (kcal/day), education (low, medium, high), physical activity (inactive, moderately inactive, moderately active, active), smoking (current, former, never), alcohol (0, 0-≤6, 6-≤12, 12-≤24, >24 grams/day), dietary fibre (continuous), glycaemic index (continuous), body-mass index (continuous), reported history of diabetes, hypertension and hyperlipidaemia, and all the macronutrients except the nutrient to be ‘replaced’ in the diet.

**Supplemental Table 5 Hazard ratios (95% CIs) of cardiovascular disease, coronary heart disease and stroke per 3% total energy intake of protein from different animal food groups**^1^

|  | Hazard ratios (95%CIs), multivariable adjusted^2^ | | |
| --- | --- | --- | --- |
| Animal protein | CVD | CHD | Stroke |
| Red meat protein | 1.01 (1.01,1.02) | 1.01 (1.01, 1.02) | 1.01 (1.00, 1.02) |
| Poultry protein | 0.99 (0.98, 1.01) | 0.99 (0.98, 1.01) | 1.00 (0.98, 1.01) |
| Processed meat protein | 1.01 (0.99, 1.03) | 1.01 (0.99, 1.04) | 1.01 (0.99, 1.04) |
| Dairy protein | 0.99 (0.98, 1.00) | 0.99 (0.99, 1.00) | 0.99 (0.97, 1.00) |
| Fish protein | 1.00 (0.98, 1.01) | 0.99 (0.98, 1.01) | 1.00 (0.98, 1.02) |
| Egg protein | 0.99 (0.97, 1.02) | 0.98 (0.95, 1.01) | 1.01 (0.98, 1.04) |

1 Proportional hazards models were used to estimate hazard ratios (HRs) and 95% confidence intervals (95% CIs) per 3% total energy intake of protein from different animal foods within each country separately, with age as the underlying time variable. Country-specific HRs (95% CIs) were combined in random-effects meta-analysis to obtain pooled effect estimates and 95% CIs.

2 The multivariable-adjusted HR included adjustment for age (years), sex (men, women), centre, energy intake (kcal/day), education (low, medium, high), physical activity (inactive, moderately inactive, moderately active, active), smoking (current, former, never), alcohol (0, 0-≤6, 6-≤12, 12-≤24, >24 grams/day), dietary fibre (continuous), glycaemic index (continuous), body-mass index (continuous), reported history of diabetes, hypertension and hyperlipidaemia,

**Supplemental Table 6 Hazard ratios (95% CIs) of cardiovascular disease, coronary heart disease and stroke by quintiles of total protein intake: EPIC-CVD Study**^1^

|  |  | Hazard ratios (95%CIs), multivariable adjusted^2^ | | | | |  |
| --- | --- | --- | --- | --- | --- | --- | --- |
|  | Substitution sources | Q1 | Q2 | Q3 | Q4 | Q5 | Per 3% energy |
| Median intake, % of total energy |  | 14.2 | 16.3 | 18.0 | 19.8 | 22.7 |  |
| CVD |  |  |  |  |  |  |  |
| N of cases/ total participants |  | 3313/ 6134 | 3078/ 5737 | 3188/ 6002 | 3209/ 6174 | 3456/ 6472 |  |
| HR, multivariable-adjusted† | No substitution | 1.0 (ref) | 0.96 (0.85, 1.09) | 0.95 (0.86, 1.05) | 0.91 (0.81, 1.02) | 1.12 (0.95, 1.32) | 1.02 (0.99, 1.06) |
|  | SFA | 1.0 (ref) | 0.94 (0.85, 1.04) | 0.94 (0.83, 1.05) | 0.89 (0.78, 1.03) | 1.12 (0.94, 1.33) | 1.05 (0.99, 1.10) |
|  | MUFA | 1.0 (ref) | 0.92 (0.81, 1.04) | 0.87 (0.77, 0.99) | 0.8 (0.69, 0.94) | 0.93 (0.75, 1.16) | 0.98 (0.92, 1.04) |
|  | PUFA | 1.0 (ref) | 0.93 (0.84, 1.04) | 0.92 (0.81, 1.05) | 0.86 (0.74, 1.01) | 1.05 (0.84, 1.30) | 1.01 (0.94, 1.09) |
|  | Carbohydrate | 1.0 (ref) | 0.96 (0.85, 1.08) | 0.94 (0.84, 1.04) | 0.89 (0.79, 1.00) | 1.09 (0.91, 1.31) | 1.02 (0.98, 1.05) |
| CHD |  |  |  |  |  |  |  |
| N of cases/ total participants |  | 1881/ 4717 | 2009/ 4773 | 2197/ 5049 | 2283/ 5318 | 2414/ 5489 |  |
| HR, multivariable-adjusted† | No substitution | 1.0 (ref) | 0.98 (0.87, 1.11) | 0.94 (0.84, 1.06) | 0.90 (0.79, 1.03) | 1.11 (0.93, 1.33) | 1.02 (0.98, 1.06) |
|  | SFA | 1.0 (ref) | 0.96 (0.86, 1.08) | 0.93 (0.81, 1.06) | 0.89 (0.76, 1.03) | 1.11 (0.91, 1.34) | 1.04 (0.98, 1.10) |
|  | MUFA | 1.0 (ref) | 0.95 (0.84, 1.07) | 0.88 (0.76, 1.03) | 0.81 (0.68, 0.96) | 0.95 (0.69, 1.3) | 0.97 (0.88, 1.07) |
|  | PUFA | 1.0 (ref) | 0.97 (0.85, 1.1) | 0.92 (0.8, 1.07) | 0.87 (0.73, 1.04) | 1.07 (0.84, 1.37) | 1.02 (0.95, 1.10) |
|  | Carbohydrate | 1.0 (ref) | 0.98 (0.86, 1.1) | 0.93 (0.83, 1.05) | 0.89 (0.78, 1.01) | 1.09 (0.89, 1.35) | 1.01 (0.97, 1.06) |
| Stroke |  |  |  |  |  |  |  |
| N of cases/ total participants |  | 1440/ 4355 | 1236/ 3987 | 1255/ 4167 | 1221/ 4284 | 1271/ 4420 |  |
| HR, multivariable-adjusted† | No substitution | 1.0 (ref) | 0.89 (0.79, 1.00) | 0.94 (0.83, 1.06) | 0.84 (0.70, 1.01) | 1.05 (0.85, 1.31) | 1.02 (0.98, 1.07) |
|  | SFA | 1.0 (ref) | 0.88 (0.78, 0.99) | 0.91 (0.79, 1.05) | 0.84 (0.71, 0.99) | 1.08 (0.88, 1.33) | 1.05 (0.99, 1.12) |
|  | MUFA | 1.0 (ref) | 0.85 (0.75, 0.97) | 0.84 (0.72, 0.98) | 0.72 (0.58, 0.89) | 0.85 (0.63, 1.15) | 0.95 (0.88, 1.03) |
|  | PUFA | 1.0 (ref) | 0.86 (0.75, 0.98) | 0.86 (0.74, 1.01) | 0.76 (0.63, 0.93) | 0.89 (0.66, 1.2) | 0.98 (0.88, 1.09) |
|  | Carbohydrate | 1.0 (ref) | 0.88 (0.78, 0.99) | 0.92 (0.81, 1.04) | 0.81 (0.67, 0.98) | 1.02 (0.81, 1.28) | 1.01 (0.97, 1.06) |

1 Proportional hazards models were used to estimate hazard ratios (HRs) and 95% confidence intervals (95% CIs) for each quintile of total protein within each country separately, with age as the underlying time variable. Country-specific HRs (95% CIs) were combined in random-effects meta-analysis to obtain pooled effect estimates and 95% CIs.

2 The multivariable-adjusted HR included adjustment for age (years), sex (men, women), centre, energy intake (kcal/day), education (low, medium, high), physical activity (inactive, moderately inactive, moderately active, active), smoking (current, former, never), alcohol (0, 0-≤6, 6-≤12, 12-≤24, >24 grams/day), dietary fibre (continuous), glycaemic index (continuous), body-mass index (continuous), reported history of diabetes, hypertension and hyperlipidaemia. In the substitution model, the association of total protein was estimated by including all macronutrients in the model as continuous variables expressed in % of total energy intake (carbohydrates, saturated fatty acids [SFA], monounsaturated fatty acids [MUFA], polyunsaturated fatty acids [PUFA], total protein) except the nutrient to be ‘replaced’ in the diet.

**Supplemental Table 7 Prospective association between dietary plant or animal-derived protein and subtypes of cardiovascular disease: EPIC-CVD Study**

|  |  | Hazard ratios (95%CIs), multivariable adjusted^1^ | | | | | |
| --- | --- | --- | --- | --- | --- | --- | --- |
|  |  | Q1 | Q2 | Q3 | Q4 | Q5 | Per 3% energy |
| **Plant-derived protein** | |  |  |  |  |  |  |
|  | Median intake, % of total energy | 3.60 | 4.71 | 5.43 | 6.19 | 7.68 |  |
|  | Fatal CVD |  |  |  |  |  |  |
|  | N of cases/ total participants | 888/ 4107 | 584/ 3760 | 447/ 3492 | 354/ 3171 | 314/ 3034 |  |
|  | HR, adjusted for age, sex, centre, energy | 1.0 (ref) | 0.79 (0.68, 0.91) | 0.80 (0.64, 1.01) | 0.77 (0.62, 0.95) | 0.83 (0.62, 1.13) | 0.83 (0.70, 0.98) |
|  | HR, multivariable-adjusted^2^ | 1.0 (ref) | 0.95 (0.80, 1.12) | 1.04 (0.85, 1.28) | 1.04 (0.82, 1.33) | 1.03 (0.76, 1.41) | 0.98 (0.77, 1.25) |
|  | Non-fatal CVD |  |  |  |  |  |  |
|  | N of cases/ total participants | 3644/ 6704 | 3178/ 6217 | 2833/ 5781 | 2458/ 5188 | 2111/ 4735 |  |
|  | HR, adjusted for age, sex, centre, energy | 1.0 (ref) | 0.90 (0.78, 1.02) | 0.87 (0.77, 0.98) | 0.91 (0.73, 1.15) | 0.92 (0.74, 1.13) | 0.90 (0.77, 1.06) |
|  | HR, multivariable-adjusted^2^ | 1.0 (ref) | 0.95 (0.85, 1.07) | 0.95 (0.85, 1.07) | 0.99 (0.81, 1.20) | 0.94 (0.78, 1.14) | 0.95 (0.84, 1.07) |
|  | Fatal CHD |  |  |  |  |  |  |
|  | N of cases/ total participants | 653/ 3883 | 446/ 3625 | 328/ 3383 | 259/ 3085 | 222/ 2948 |  |
|  | HR, adjusted for age, sex, centre, energy | 1.0 (ref) | 0.82 (0.71, 0.96) | 0.83 (0.63, 1.10) | 0.84 (0.60, 1.17) | 0.90 (0.70, 1.16) | 0.86 (0.73, 1.02) |
|  | HR, multivariable-adjusted^2^ | 1.0 (ref) | 1.05 (0.86, 1.27) | 1.17 (0.89, 1.54) | 1.28 (0.91, 1.81) | 1.31 (0.88, 1.94) | 1.12 (0.89, 1.41) |
|  | Non-fatal CHD |  |  |  |  |  |  |
|  | N of cases/ total participants | 2177/ 5327 | 1993/ 5090 | 1833/ 4827 | 1672/ 4443 | 1483/ 4141 |  |
|  | HR, adjusted for age, sex, centre, energy | 1.0 (ref) | 0.90 (0.77, 1.06) | 0.87 (0.74, 1.02) | 0.95 (0.73, 1.23) | 0.88 (0.69, 1.13) | 0.89 (0.75, 1.06) |
|  | HR, multivariable-adjusted^2^ | 1.0 (ref) | 0.96 (0.82, 1.12) | 0.95 (0.83, 1.09) | 1.03 (0.84, 1.26) | 0.90 (0.69, 1.17) | 0.93 (0.81, 1.05) |
|  | Fatal stroke |  |  |  |  |  |  |
|  | N of cases/ total participants | 250/ 3505 | 149/ 3351 | 131/ 3190 | 102/ 2944 | 107/ 2842 |  |
|  | HR, adjusted for age, sex, centre, energy | 1.0 (ref) | 0.67 (0.53, 0.85) | 0.75 (0.54, 1.04) | 0.66 (0.49, 0.88) | 0.72 (0.43, 1.21) | 0.77 (0.60, 0.97) |
|  | HR, multivariable-adjusted^2^ | 1.0 (ref) | 0.69 (0.53, 0.91) | 0.80 (0.58, 1.09) | 0.66 (0.45, 0.96) | 0.69 (0.43, 1.13) | 0.67 (0.44, 1.03) |
|  | Non-fatal stroke |  |  |  |  |  |  |
|  | N of cases/ total participants | 1661/ 4832 | 1343/ 4479 | 1148/ 4161 | 910/ 3713 | 754/ 3461 |  |
|  | HR, adjusted for age, sex, centre, energy | 1.0 (ref) | 0.87 (0.78, 0.96) | 0.88 (0.78, 0.98) | 0.84 (0.69, 1.03) | 0.94 (0.80, 1.12) | 0.91 (0.78, 1.07) |
|  | HR, multivariable-adjusted^2^ | 1.0 (ref) | 0.95 (0.84, 1.07) | 0.98 (0.85, 1.13) | 0.94 (0.78, 1.12) | 1.00 (0.81, 1.23) | 0.96 (0.83, 1.12) |
|  |  |  |  |  |  |  |  |
| **Animal-derived protein** | |  |  |  |  |  |  |
|  | Median intake, % of total energy | 6.66 | 9.28 | 11.1 | 13.0 | 16.7 |  |
|  | Fatal CVD |  |  |  |  |  |  |
|  | N of cases/ total participants | 536/ 3414 | 521/ 3375 | 523/ 3452 | 504/ 3632 | 503/ 3691 |  |
|  | HR, adjusted for age, sex, centre, energy | 1.0 (ref) | 0.83 (0.68, 1.01) | 0.88 (0.75, 1.04) | 0.77 (0.65, 0.93) | 1.17 (0.90, 1.51) | 1.06 (1.01, 1.11) |
|  | HR, multivariable-adjusted^2^ | 1.0 (ref) | 0.78 (0.65, 0.93) | 0.79 (0.66, 0.95) | 0.68 (0.55, 0.83) | 0.94 (0.70, 1.26) | 0.99 (0.93, 1.05) |
|  | Non-fatal CVD |  |  |  |  |  |  |
|  | N of cases/ total participants | 2381/ 5201 | 2614/ 5363 | 2902/ 5729 | 3099/ 6084 | 3228/ 6248 |  |
|  | HR, adjusted for age, sex, centre, energy | 1.0 (ref) | 0.98 (0.89, 1.08) | 1.03 (0.93, 1.14) | 1.02 (0.92, 1.13) | 1.23 (1.05, 1.43) | 1.07 (1.04, 1.10) |
|  | HR, multivariable-adjusted^2^ | 1.0 (ref) | 0.94 (0.85, 1.04) | 0.96 (0.86, 1.06) | 0.93 (0.83, 1.04) | 1.08 (0.92, 1.26) | 1.02 (0.99, 1.05) |
|  | Fatal CHD |  |  |  |  |  |  |
|  | N of cases/ total participants | 392/ 3275 | 385/ 3246 | 388/ 3323 | 356/ 3496 | 387/ 3584 |  |
|  | HR, adjusted for age, sex, centre, energy | 1.0 (ref) | 0.85 (0.64, 1.13) | 0.88 (0.73, 1.06) | 0.74 (0.61, 0.91) | 1.25 (0.98, 1.59) | 1.08 (1.02, 1.14) |
|  | HR, multivariable-adjusted^2^ | 1.0 (ref) | 0.78 (0.62, 0.97) | 0.77 (0.62, 0.95) | 0.64 (0.50, 0.80) | 0.98 (0.74, 1.31) | 0.99 (0.93, 1.06) |
|  | Non-fatal CHD |  |  |  |  |  |  |
|  | N of cases/ total participants | 1547/ 4405 | 1682/ 4474 | 1859/ 4731 | 1982/ 5037 | 2088/ 5184 |  |
|  | HR, adjusted for age, sex, centre, energy | 1.0 (ref) | 1.00 (0.90, 1.11) | 1.05 (0.93, 1.19) | 1.05 (0.93, 1.27) | 1.26 (1.11, 1.43) | 1.07 (1.04, 1.10) |
|  | HR, multivariable-adjusted^2^ | 1.0 (ref) | 0.97 (0.87, 1.09) | 0.98 (0.86, 1.11) | 0.97 (0.85, 1.10) | 1.10 (0.96, 1.27) | 1.02 (0.98, 1.05) |
|  | Fatal stroke |  |  |  |  |  |  |
|  | N of cases/ total participants | 152/ 3054 | 150/ 3032 | 145/ 3099 | 162/ 3305 | 130/ 3342 |  |
|  | HR, adjusted for age, sex, centre, energy | 1.0 (ref) | 0.84 (0.65, 1.09) | 0.94 (0.72, 1.23) | 0.97 (0.74, 1.28) | 1.14 (0.81, 1.60) | 1.04 (0.96, 1.12) |
|  | HR, multivariable-adjusted^2^ | 1.0 (ref) | 0.83 (0.63, 1.08) | 0.91 (0.69, 1.20) | 0.91 (0.68, 1.23) | 1.07 (0.76, 1.51) | 1.01 (0.92, 1.11) |
|  | Non-fatal stroke |  |  |  |  |  |  |
|  | N of cases/ total participants | 981/ 3846 | 1065/ 3906 | 1204/ 4108 | 1263/ 4347 | 1303/ 4439 |  |
|  | HR, adjusted for age, sex, centre, energy | 1.0 (ref) | 0.91 (0.81, 1.03) | 0.98 (0.87, 1.11) | 0.95 (0.82, 1.10) | 1.12 (0.88, 1.44) | 1.06 (1.01, 1.11) |
|  | HR, multivariable-adjusted^2^ | 1.0 (ref) | 0.88 (0.77, 0.99) | 0.92 (0.81, 1.05) | 0.86 (0.75, 0.99) | 1.00 (0.80, 1.25) | 1.02 (0.98, 1.06) |
|  |  |  |  |  |  |  |  |
| **Total protein** | |  |  |  |  |  |  |
|  | Median intake, % of total energy | 13.8 | 16.3 | 18.0 | 19.8 | 23.3 |  |
|  | Fatal CVD |  |  |  |  |  |  |
|  | N of cases/ total participants | 675/ 3613 | 539/ 3304 | 490/ 3451 | 406/ 3528 | 477/ 3668 |  |
|  | HR, adjusted for age, sex, centre, energy | 1.0 (ref) | 0.90 (0.65, 1.23) | 0.91 (0.65, 1.27) | 0.76 (0.59, 0.98) | 1.19 (0.87, 1.64) | 1.05 (1.00, 1.11) |
|  | HR, multivariable-adjusted^2^ | 1.0 (ref) | 0.89 (0.64, 1.23) | 0.88 (0.64, 1.21) | 0.66 (0.50, 0.86) | 1.04 (0.77, 1.40) | 1.01 (0.95, 1.08) |
|  | Non-fatal CVD |  |  |  |  |  |  |
|  | N of cases/ total participants | 2809/ 5664 | 2668/ 5353 | 2792/ 5625 | 2889/ 5872 | 3066/ 6111 |  |
|  | HR, adjusted for age, sex, centre, energy | 1.0 (ref) | 0.96 (0.87, 1.05) | 1.00 (0.90, 1.12) | 1.03 (0.92, 1.14) | 1.26 (1.07, 1.48) | 1.07 (1.04, 1.10) |
|  | HR, multivariable-adjusted^2^ | 1.0 (ref) | 0.96 (0.86, 1.08) | 0.96 (0.86, 1.06) | 0.95 (0.84, 1.06) | 1.13 (0.96, 1.33) | 1.03 (0.99, 1.06) |
|  | Fatal CHD |  |  |  |  |  |  |
|  | N of cases/ total participants | 506/ 3452 | 395/ 3166 | 344/ 3312 | 308/ 3437 | 355/ 3557 |  |
|  | HR, adjusted for age, sex, centre, energy | 1.0 (ref) | 0.90 (0.60, 1.36) | 0.95 (0.64, 1.42) | 0.90 (0.62, 1.29) | 1.30 (0.90, 1.87) | 1.07 (1.00, 1.14) |
|  | HR, multivariable-adjusted^2^ | 1.0 (ref) | 0.88 (0.59, 1.30) | 0.85 (0.61, 1.19) | 0.70 (0.51, 0.96) | 1.01 (0.72, 1.43) | 1.02 (0.94, 1.09) |
|  | Non-fatal CHD |  |  |  |  |  |  |
|  | N of cases/ total participants | 1677/ 4584 | 1705/ 4423 | 1806/ 4700 | 1881/ 4927 | 2089/ 5194 |  |
|  | HR, adjusted for age, sex, centre, energy | 1.0 (ref) | 0.98 (0.88, 1.08) | 1.02 (0.92, 1.14) | 1.02 (0.90, 1.14) | 1.24 (1.04, 1.49) | 1.07 (1.03, 1.10) |
|  | HR, multivariable-adjusted^2^ | 1.0 (ref) | 0.98 (0.88, 1.10) | 0.97 (0.86, 1.09) | 0.94 (0.83, 1.08) | 1.11 (0.92, 1.34) | 1.02 (0.98, 1.06) |
|  | Fatal stroke |  |  |  |  |  |  |
|  | N of cases/ total participants | 179/ 3153 | 154/ 2944 | 156/ 3134 | 112/ 3246 | 138/ 3355 |  |
|  | HR, adjusted for age, sex, centre, energy | 1.0 (ref) | 0.80 (0.56, 1.14) | 0.95 (0.69, 1.31) | 0.72 (0.53, 1.97) | 1.00 (0.65, 1.52) | 1.04 (0.94, 1.15) |
|  | HR, multivariable-adjusted^2^ | 1.0 (ref) | 0.78 (0.53, 1.16) | 0.99 (0.74, 1.32) | 0.67 (0.49, 0.92) | 1.03 (0.70, 1.51) | 1.03 (0.93, 1.14) |
|  | Non-fatal stroke |  |  |  |  |  |  |
|  | N of cases/ total participants | 1304/ 4225 | 1110/ 3867 | 1126/ 4043 | 1129/ 4203 | 1147/ 4308 |  |
|  | HR, adjusted for age, sex, centre, energy | 1.0 (ref) | 0.91 (0.01, 1.03) | 0.96 (0.85, 1.08) | 0.94 (0.77, 1.15) | 1.18 (0.96, 1.43) | 1.06 (1.02, 1.10) |
|  | HR, multivariable-adjusted^2^ | 1.0 (ref) | 0.89 (0.79, 1.00) | 0.93 (0.82, 1.05) | 0.87 (0.73, 1.04) | 1.08 (0.88, 1.32) | 1.03 (0.98, 1.07) |

1 Proportional hazards models were used to estimate hazard ratios (HRs) and 95% confidence intervals (95% CIs) for quintile 2-5 (Q2-Q5) compared with Q1 and per 3% energy increase within each country separately, with age as the underlying time variable. Country-specific HRs (95% CIs) were combined in random-effects meta-analysis to obtain pooled effect estimates and 95% CIs.

2 The multivariable-adjusted HR included adjustment for age (years), sex (men, women), centre, energy intake (kcal/day), education (low, medium, high), physical activity (inactive, moderately inactive, moderately active, active), smoking (current, former, never), alcohol (0, 0-≤6, 6-≤12, 12-≤24, >24 grams/day), dietary fibre (continuous), glycaemic index (continuous), body-mass index (continuous), reported history of diabetes, hypertension and hyperlipidaemia. No specific replacement of energy from other macronutrients was performed in the models.

**Supplemental Table 8 Prospective association of dietary protein and subtypes of cardiovascular disease in isocaloric substitution analysis replacing 3% higher energy intake from plant, animal or total protein with energy from other sources: EPIC-CVD Study^1^**

| Substitution sources | Hazard ratio (95% confidence intervals), multivariable-adjusted^2^ | | | | | |
| --- | --- | --- | --- | --- | --- | --- |
|  | Fatal CVD | Non-fatal CVD | Fatal CHD | Non-fatal CHD | Fatal stroke | Non-fatal stroke |
| **Plant-derived protein** |  |  |  |  |  |  |
| SFA | 1.07 (0.82, 1.40) | 1.01 (0.87, 1.17) | 1.26 (0.95, 1.67) | 0.97 (0.83, 1.13) | 0.72 (0.41, 1.24) | 1.02 (0.87, 1.21) |
| MUFA | 1.01 (0.72, 1.42) | 0.94 (0.77, 1.16) | 1.13 (0.84, 1.53) | 0.92 (0.72, 1.18) | 0.77 (0.42, 1.41) | 0.94 (0.78, 1.14) |
| PUFA | 1.06 (0.77, 1.44) | 0.99 (0.85, 1.15) | 1.19 (0.85, 1.66) | 0.98 (0.82, 1.16) | 0.71 (0.39, 1.31) | 0.94 (0.78, 1.14) |
| Carbohydrate | 1.04 (0.77, 1.40) | 0.99 (0.84, 1.17) | 1.18 (0.91, 1.55) | 0.95 (0.80, 1.14) | 0.70 (0.40, 1.24) | 0.99 (0.83, 1.17) |
| Animal protein | 1.03 (0.77, 1.38) | 0.96 (0.83, 1.12) | 1.18 (0.90, 1.53) | 0.93 (0.79, 1.09) | 0.71 (0.43, 1.20) | 0.97 (0.82, 1.14) |
|  |  |  |  |  |  |  |
| **Animal-derived protein** |  |  |  |  |  |  |
| SFA | 1.02 (0.93, 1.13) | 1.06 (1.00, 1.11) | 1.02 (0.91, 1.15) | 1.05 (0.99, 1.12) | 1.07 (0.91, 1.25) | 1.06 (0.99, 1.13) |
| MUFA | 0.98 (0.86, 1.12) | 0.99 (0.91, 1.07) | 0.96 (0.82, 1.11) | 0.99 (0.88, 1.11) | 1.06 (0.87, 1.31) | 0.95 (0.88, 1.04) |
| PUFA | 0.99 (0.87, 1.13) | 1.02 (0.95, 1.09) | 0.99 (0.85, 1.15) | 1.04 (0.97, 1.12) | 1.02 (0.83, 1.24) | 0.96 (0.88, 1.05) |
| Carbohydrate | 1.01 (0.95, 1.08) | 1.03 (0.99, 1.07) | 1.01 (0.94, 1.09) | 1.03 (0.98, 1.08) | 1.06 (0.95, 1.17) | 1.02 (0.98, 1.07) |
| Plant protein | 0.96 (0.72, 1.27) | 1.02 (0.87, 1.19) | 0.84 (0.65, 1.08) | 1.05 (0.88, 1.25) | 1.33 (0.78, 2.28) | 1.02 (0.86, 1.20) |
|  |  |  |  |  |  |  |
| **Total protein** |  |  |  |  |  |  |
| SFA | 1.04 (0.94, 1.14) | 1.04 (0.99, 1.10) | 1.05 (0.94, 1.18) | 1.04 (0.97, 1.10) | 1.06 (0.92, 1.23) | 1.05 (0.98, 1.12) |
| MUFA | 0.96 (0.84, 1.08) | 0.98 (0.92, 1.05) | 0.93 (0.81, 1.08) | 0.98 (0.88, 1.08) | 1.00 (0.81, 1.24) | 0.95 (0.87, 1.03) |
| PUFA | 1.00 (0.88, 1.13) | 1.01 (0.95, 1.08) | 0.99 (0.85, 1.14) | 1.03 (0.96, 1.11) | 1.04 (0.86, 1.27) | 0.96 (0.88, 1.06) |
| Carbohydrate | 1.00 (0.94, 1.07) | 1.02 (0.98, 1.05) | 1.00 (0.93, 1.08) | 1.01 (0.97, 1.06) | 1.03 (0.91, 1.15) | 1.02 (0.97, 1.06) |

1 Proportional hazards models were used to estimate hazard ratios (HRs) and 95% confidence intervals (95% CIs) for each 3% higher energy intake from plant- and animal- derived protein or total protein within each country separately, with age as the underlying time variable. Country-specific HRs (95% CIs) were combined in random-effects meta-analysis to obtain pooled effect estimates and 95% CIs. The total number of fatal CVD, CHD and stroke cases was 2587, 1908 and 739, respectively; and number of non-fatal CVD, CHD and stroke was 14224, 9158 and 5816, respectively.

2 The multivariable-adjusted HR included adjustment for age (years), sex (men, women), centre, energy intake (kcal/day), education (low, medium, high), physical activity (inactive, moderately inactive, moderately active, active), smoking (current, former, never), alcohol (0, 0-≤6, 6-≤12, 12-≤24, >24 grams/day), dietary fibre (continuous), glycaemic index (continuous), body-mass index (continuous), reported history of diabetes, hypertension and hyperlipidaemia, and all the macronutrients except the nutrient to be ‘replaced’ in the diet.

**Supplemental Figure 1. Study flowchart**

**
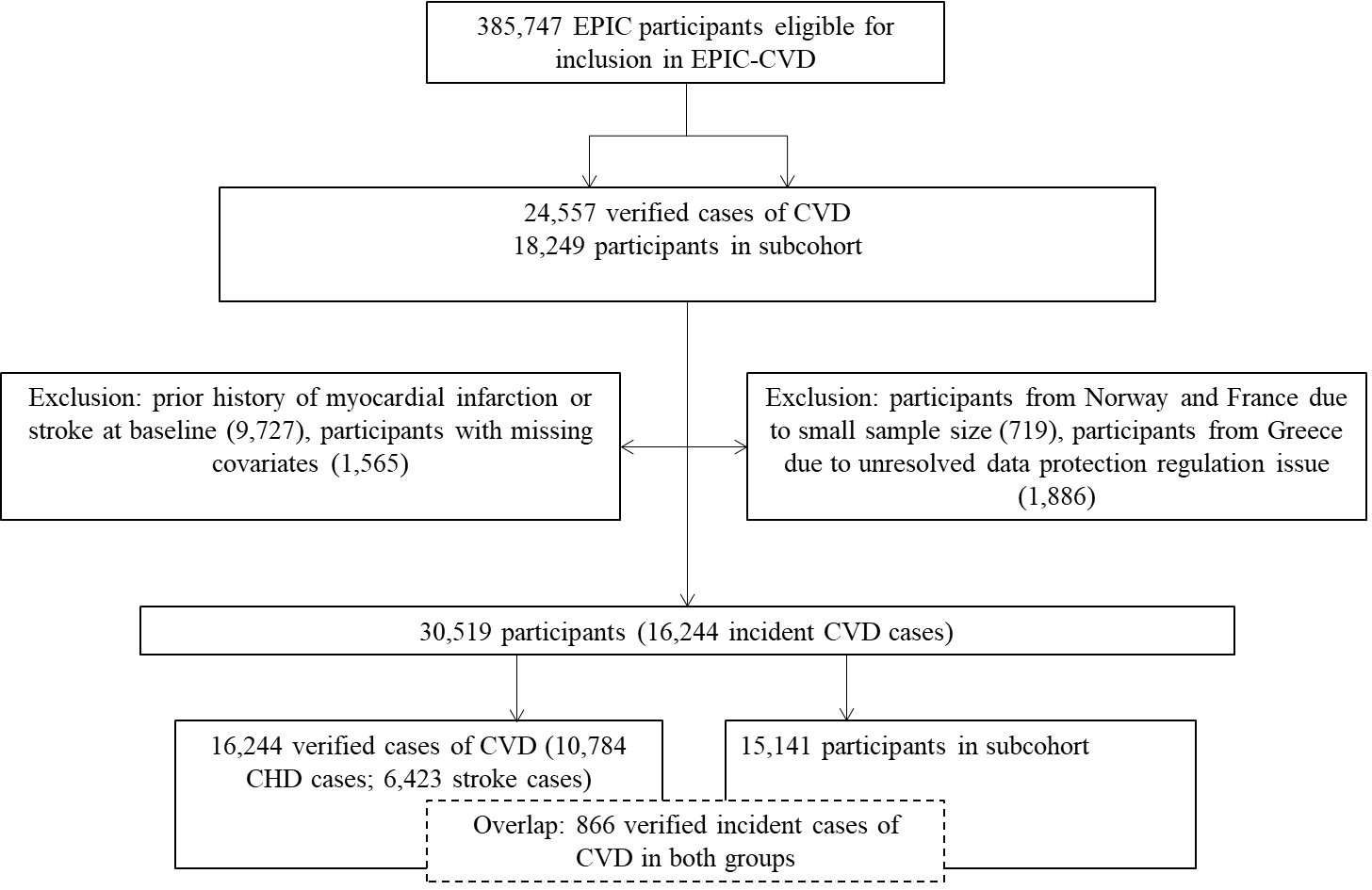
**

**Supplemental Figure 2. Distribution of observed dietary protein intake in the subcohort: EPIC-CVD Study.** Red lines indicate interquartile range of the distribution in the overall subcohort. Grey boxes indicate median (interquartile range) for the dietary protein intake.


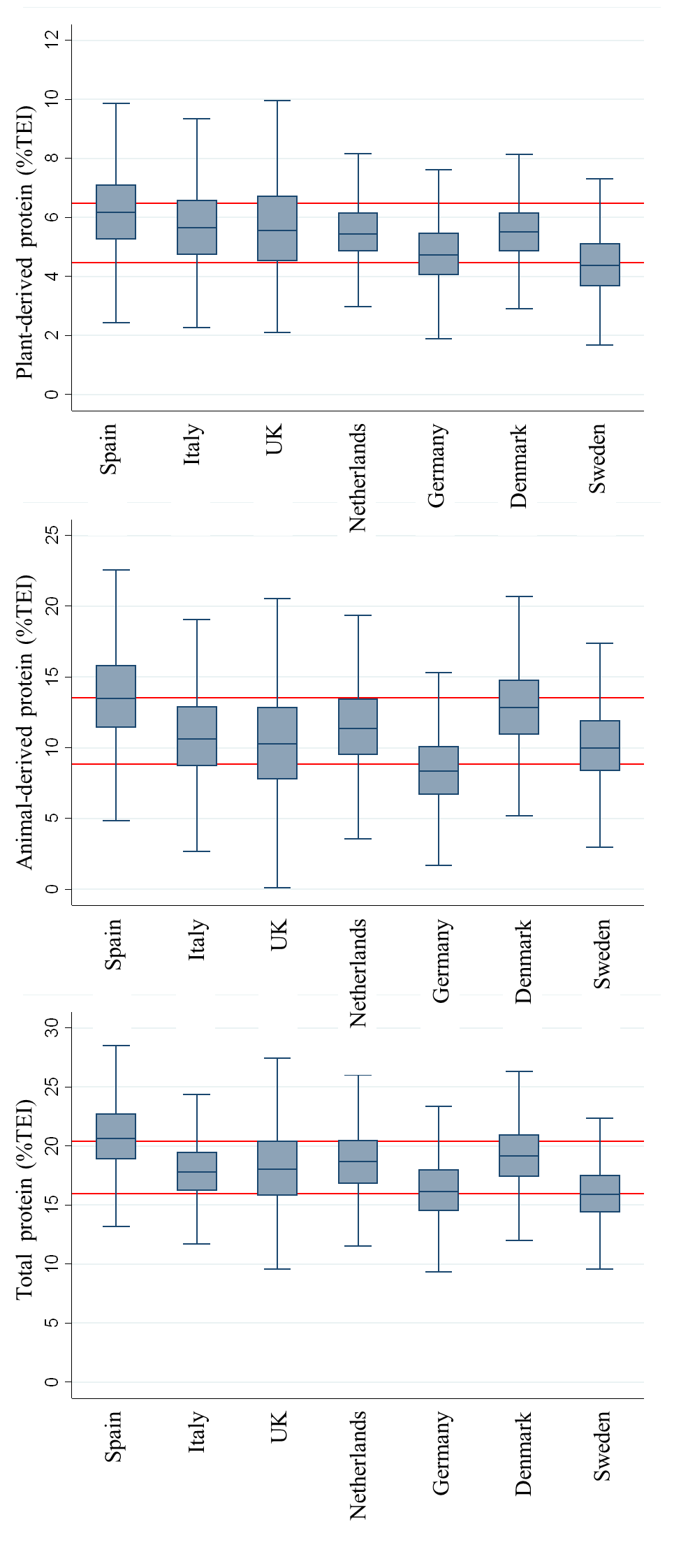


**Supplemental Figure 3 Prospective association between dietary protein and total cardiovascular disease, coronary heart disease and stroke stratified by country: EPIC-CVD Study.** Proportional hazards models were used to estimate multivariable-adjusted hazard ratios (HRs) of total CVD, CHD and stroke for each 3% higher energy intake from plant- and animal- derived protein or total protein within each country separately, with age as the underlying time variable. Country-specific HRs (95% CIs) were combined in random-effects meta-analysis to obtain pooled effect estimates and 95% CIs. No specific replacement of energy from other macronutrients was performed in the models. The adjusted covariates were described in details in the text.

**
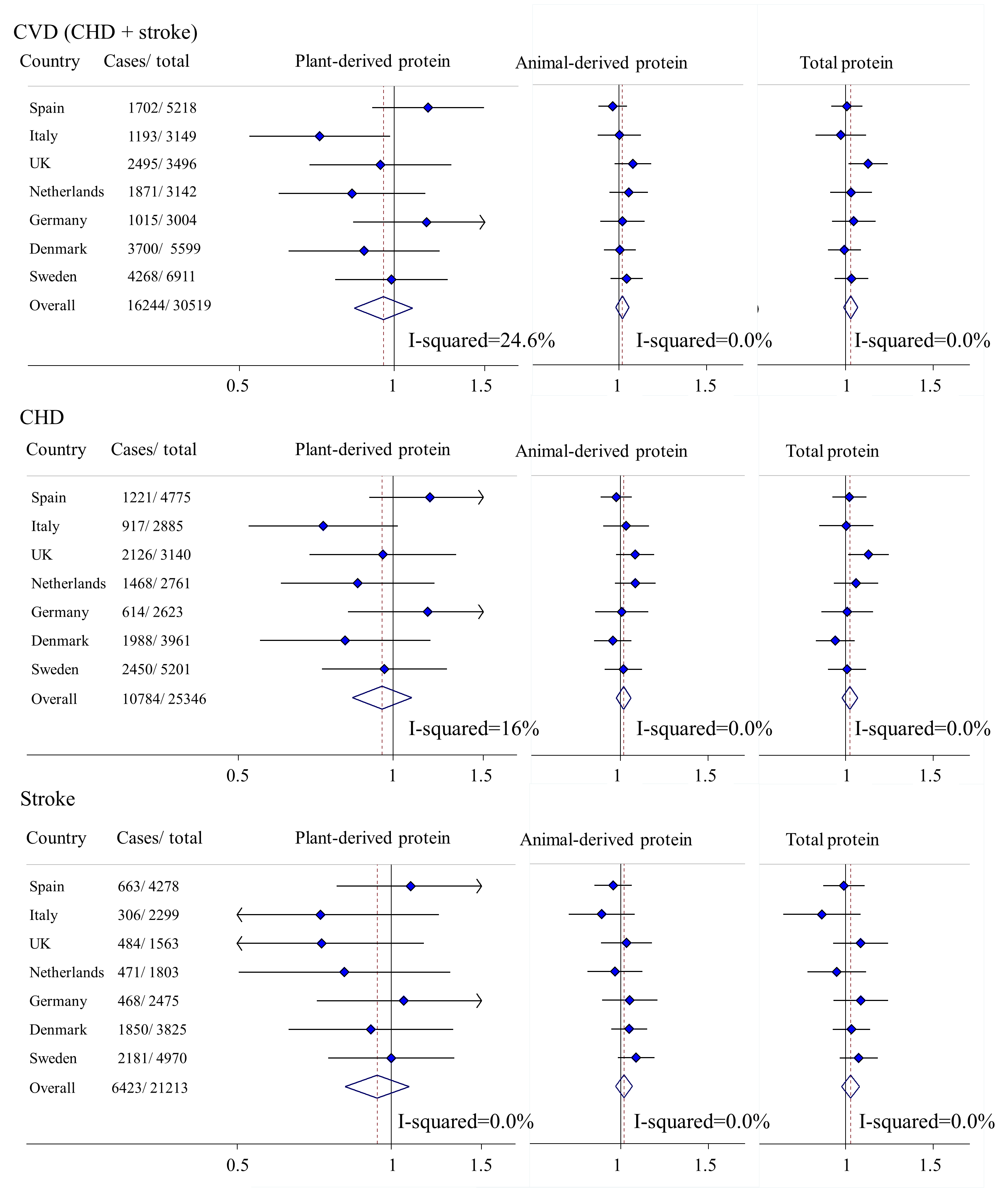
**

**Supplemental Figure 4 Prospective association between protein consumption and cardiovascular diseases in isocaloric substitution analyses replacing 3% energy intake from different macronutrients with plant- or animal-derived protein: EPIC-CVD Study.** Proportional hazards models were used to estimate hazard ratios (HRs) and 95% confidence intervals (CIs) for each 3% higher energy intake from plant- or animal- derived protein within each country separately, with age as the underlying time variable. Country-specific HRs (95% CIs) were combined in random-effects meta-analysis to obtain pooled effect estimates and 95% CIs. The total number of CVD, CHD and stroke cases was 16925, 11191 and 6745, respectively, and 16902 participants were included in the subcohort. Isocaloric substitution models were used in the analyses, where the association of plant- or animal-derived protein with CVD endpoints was estimated by including all macronutrients in the model as continuous variables expressed in % of total energy intake (carbohydrates, saturated fat, monounsaturated fat, polyunsaturated fat, mixed/unknown origin protein and plant- or animal-derived protein) except the nutrient to be ‘replaced’ in the diet.

**
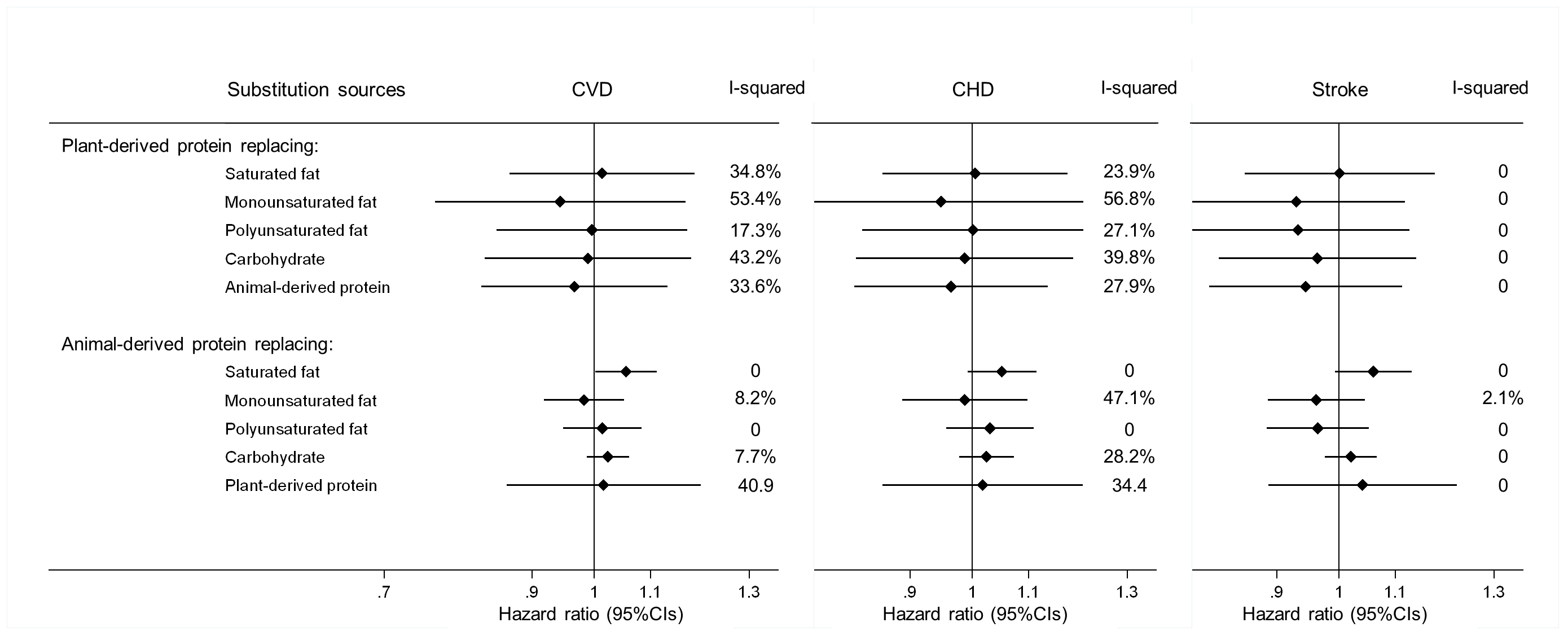
**

**Supplemental Figure 5 Hazard ratios (95%CIs) of total cardiovascular disease, coronary heart disease and stroke for each 3% higher energy intake from plant- derived protein to replace energy from other macronutrients stratified by country: EPIC-CVD Study.** Proportional hazards models were used to estimate multivariable-adjusted hazard ratios (HRs) of total CVD, CHD and stroke for each 3% higher energy intake from plant- derived protein to replace energy from other macronutrients within each country separately, with age as the underlying time variable. Country-specific HRs (95% CIs) were combined in random-effects meta-analysis to obtain pooled effect estimates and 95% CIs. No specific replacement of energy from other macronutrients was performed in the models. The adjusted covariates were described in details in the text.


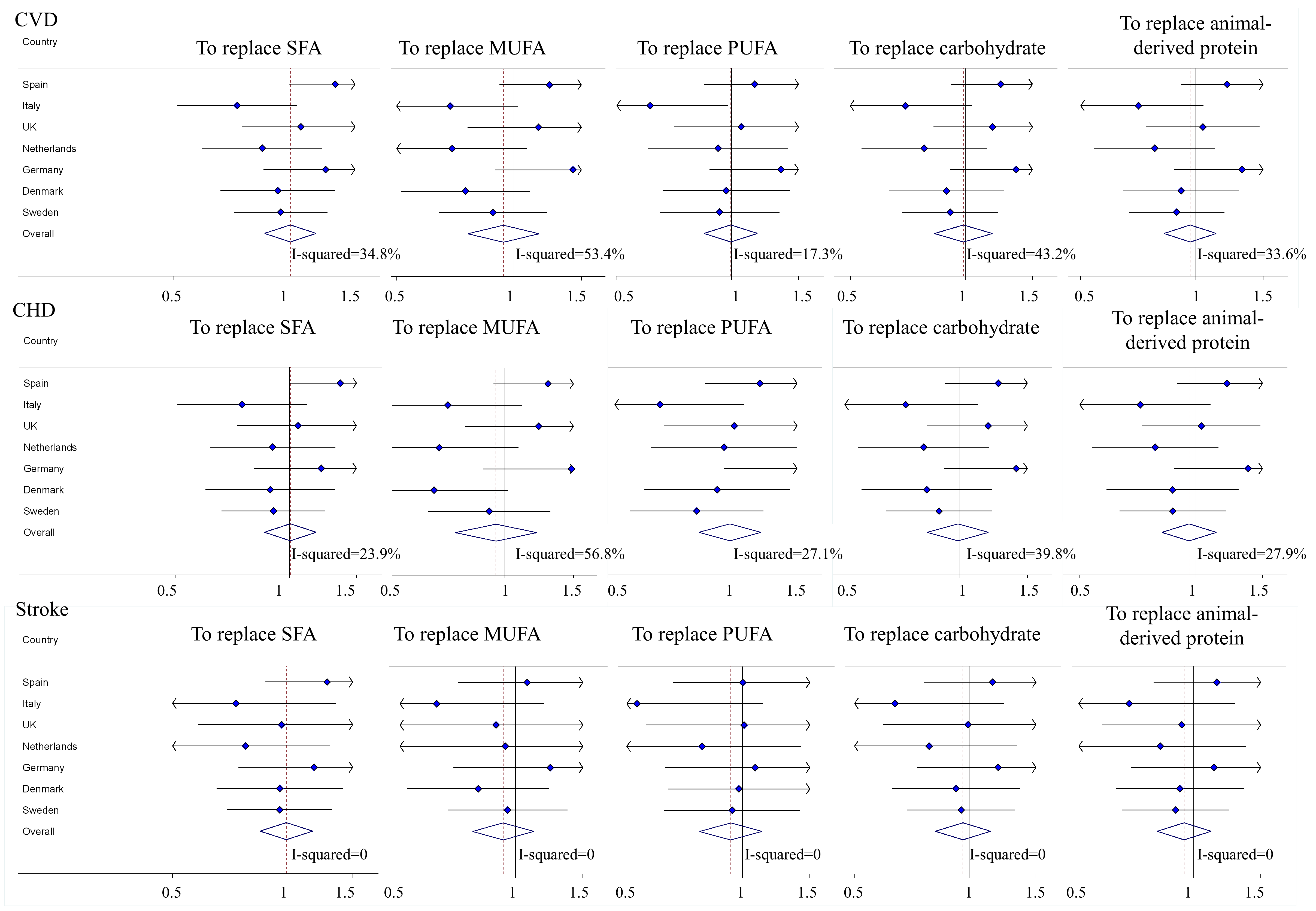


**Supplemental Figure 6 Hazard ratios (95%CIs) of total cardiovascular disease, coronary heart disease and stroke for each 3% higher energy intake from animal- derived protein to replace energy from other macronutrients stratified by country: EPIC-CVD Study.** Proportional hazards models were used to estimate multivariable-adjusted hazard ratios (HRs) of total CVD, CHD and stroke for each 3% higher energy intake from animal- derived protein to replace energy from other macronutrients within each country separately, with age as the underlying time variable. Country-specific HRs (95% CIs) were combined in random-effects meta-analysis to obtain pooled effect estimates and 95% CIs. No specific replacement of energy from other macronutrients was performed in the models. The adjusted covariates were described in details in the text.


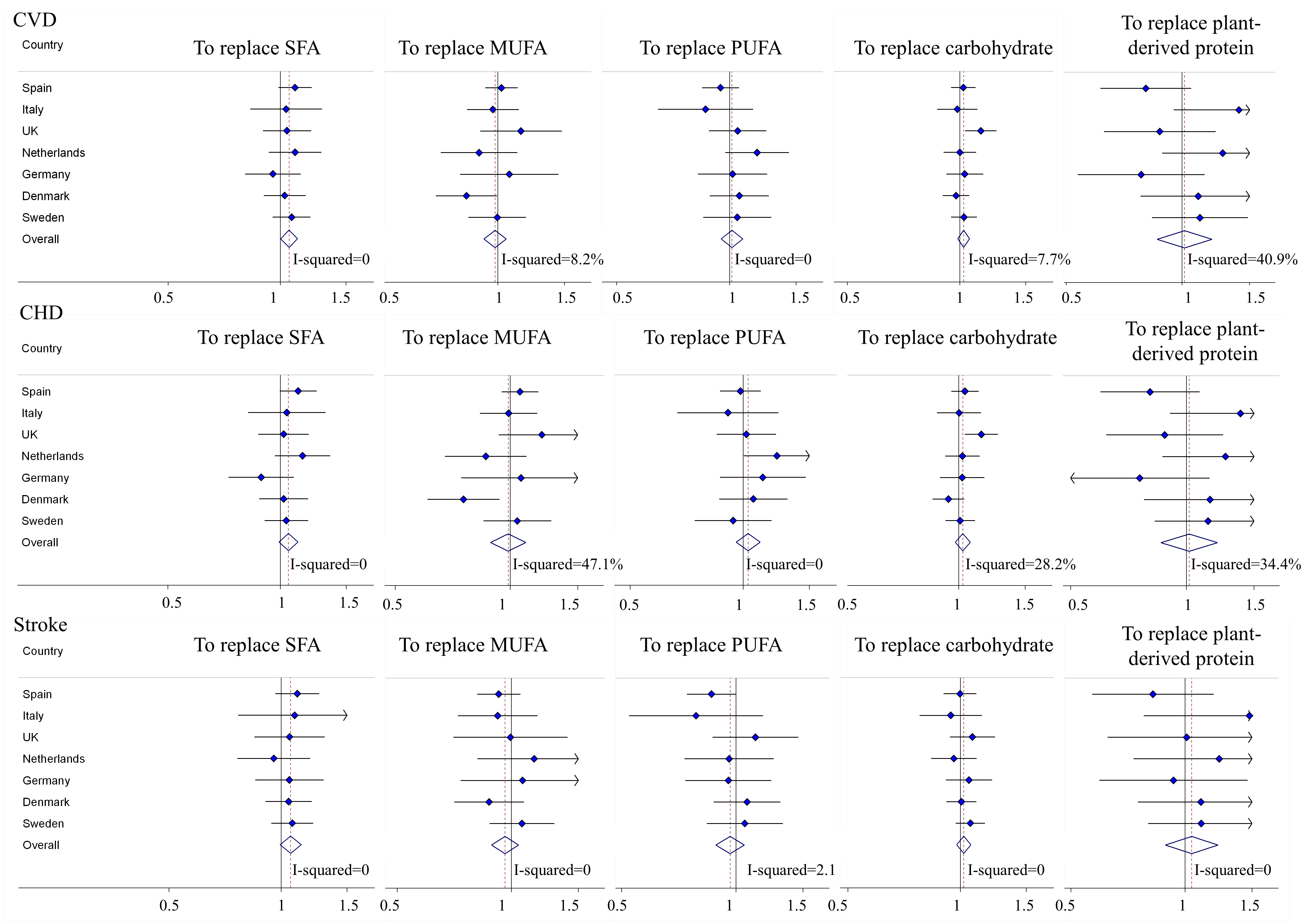


**Supplemental Figure 7 Hazard ratio (95%CI) of total cardiovascular disease, coronary heart disease and stroke for each 3% higher energy intake from total protein to replace energy from other macronutrients stratified by country: EPIC-CVD Study.** Proportional hazards models were used to estimate multivariable-adjusted hazard ratios (HRs) of total CVD, CHD and stroke for each 3% higher energy intake from total protein to replace energy from other macronutrients within each country separately, with age as the underlying time variable. Country-specific HRs (95% CIs) were combined in random-effects meta-analysis to obtain pooled effect estimates and 95% CIs. No specific replacement of energy from other macronutrients was performed in the models. The adjusted covariates were described in detail in the text.

**
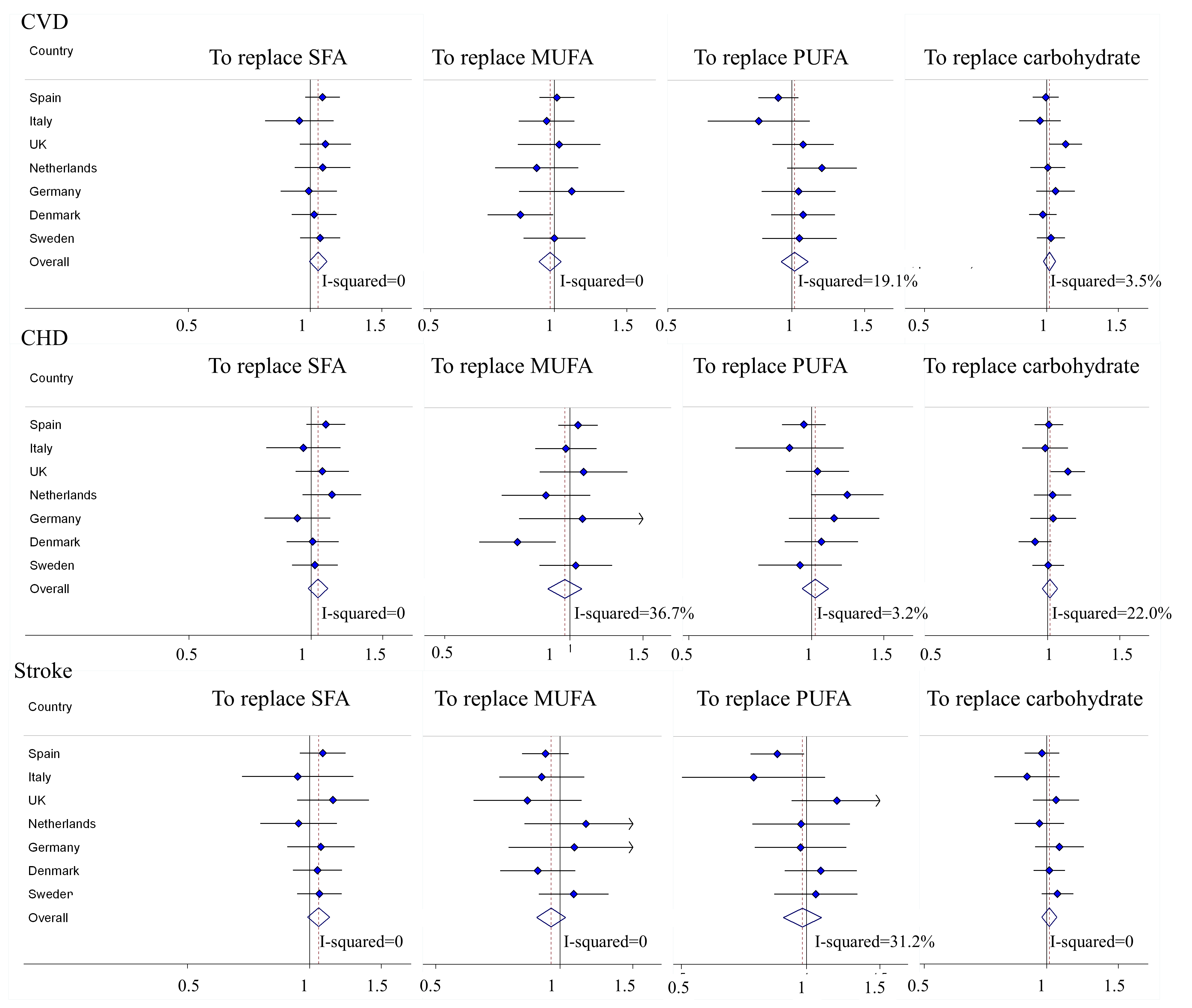
**
